# Structural insights into *ortho*-aminophenol oxidases: kinetic and crystallographic characterization of *Sm*NspF and *Sg*GriF

**DOI:** 10.1039/d5qi02495a

**Published:** 2026-01-30

**Authors:** Hoa Le Xuan, Annette Rompel

**Affiliations:** a Universität Wien, Fakultät für Chemie, Institut für Biophysikalische Chemie Josef-Holaubek Platz 2 1090 Vienna Austria annette.rompel@univie.ac.at https://www.bpc.univie.ac.at; b Universität Wien, Vienna Doctoral School in Chemistry (DoSChem) Währinger Straße 42 1090 Vienna Austria https://doschem.univie.ac.at

## Abstract

Actinobacteria-derived *o*-aminophenol oxidases (AOs) represent a largely unexplored subclass of type-III copper enzymes with catalytic properties distinct from tyrosinases and catechol oxidases. The determination of the first crystal structure of an AO (*Sm*NspF) displays unique loop insertions and important second-sphere amino acids in vicinity of the binuclear copper center. The substrate-guiding effect of the second activity controller (His_B2+1_) influences the binding affinity for carboxyl-containing substrates in the AOs *Sm*NspF and *Sg*GriF. Thus, kinetic investigations reveal both overlapping and distinct substrate preferences for *Sm*NspF and *Sg*GriF: while both enzymes oxidize monophenols, *o*-aminophenols, and *o*-diphenols, they do so at significantly different reaction rates. *Sm*NspF preferentially oxidizes carboxylated substrates such as 3,4-dihydroxybenzoic acid and 3-amino-4-hydroxybenzoic acid, whereas *Sg*GriF exhibits higher activity toward *para*-methylated analogs, including 4-methylcatechol and 2-amino-4-methylphenol. Remarkably, both enzymes display enzymatic activities beyond the known AO reactivity spectrum by oxidizing 2-aminoresorcinol and *o*-phenylenediamine, which underlies the high versatility of the binuclear copper center. Altogether, these findings provide a structural basis for AO's enzymatic activity and broaden the known catalytic spectrum, which enables the prediction of catalytic properties in type-III copper proteins based on their amino acid sequence.

## Abstract 2

*Sm*NspF and *Sg*GriF (type-III copper enzymes) accept phenols, *o*-aminophenols, and *o*-phenylenediamines but differ in reaction rates due to residue variations near the active center that affect substrate binding, particularly for carboxylated substrates.

## Graphical abstract

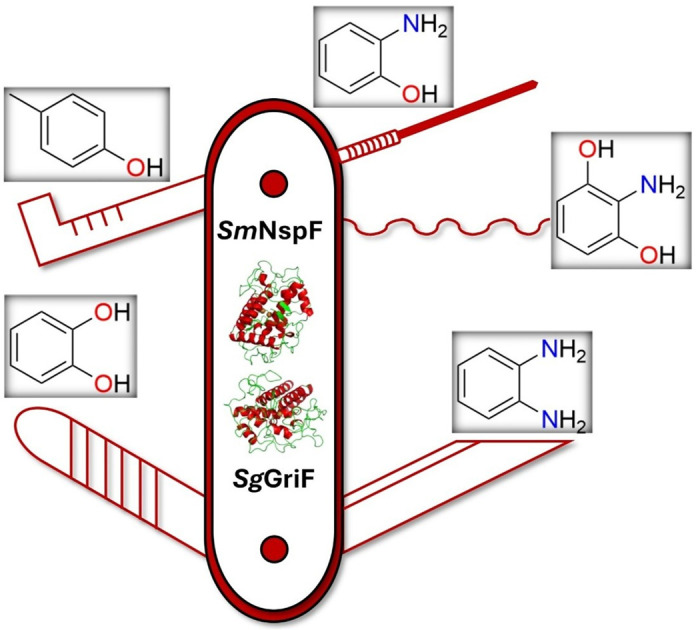

## Introduction

Actinobacteria of the genus *Streptomyces* are renowned for their exceptional metabolic diversity and their ability to produce a wide range of bioactive natural products, including antibiotics, antifungals, immunosuppressants, antiparasitic agents and pigments.^1–5^ One example from this genus is *Streptomyces murayamaensis*, which produces the enzyme *o*-aminophenol oxidase (AO) *Sm*NspF (Uniprot ID: D6RTB9), and another example is *Streptomyces griseus*, which produces the AO *Sg*GriF (Uniprot ID: B1VTI5).^6,7^*o*-Aminophenol oxidases (AOs; EC 1.10.3.4) belong to the type-III copper enzyme family, a widespread group of metalloenzymes characterized by a binuclear copper center, each coordinated by three histidine residues. Type-III copper enzymes occur in all domains of life, spanning archaea, bacteria, fungi, plants, and animals.^8–11^ In all subclasses of type-III copper proteins, the active site is proposed to bind molecular oxygen in a symmetric side-on bridging configuration (μ-η^2^:η^2^), which facilitates the activation of O_2_ for subsequent substrate oxidation.^12–14^ The best-characterized representatives of type-III copper proteins include catechol oxidases (EC 1.10.3.1), which oxidize *o*-diphenols to *o*-quinones, and tyrosinases (TYR; EC 1.14.18.1), which possess both monophenolase activity (hydroxylation of monophenols to *o*-diphenols) and diphenolase activity (Fig. S1).^12,13^ The relatively underexplored AOs have been reported to exhibit both monophenolase and diphenolase activities, suggesting enzymatic overlap with TYRs.^14^ However, AOs uniquely catalyze the oxidation of *o*-aminophenols to nitrosophenols (nitroso-forming activity), a reaction not observed in catechol oxidases or TYRs (Fig. S1).^11,14^ The structural basis for the functional divergence between AOs and TYRs has been attributed to a key active-site residue (activity selector) with AOs featuring a conserved asparagine and TYRs a conserved tyrosine at a position near the active center.^15^ Substituting Asn43 in the *Sg*GriF with the isoleucine present at the homologous position in TYR *Sz*TYR, a tyrosinase originated from *Streptomyces* sp. ZL-24, abolishes the nitroso-forming activity, whereas introducing an asparagine in place of the homologous isoleucine in *Sz*TYRs confers this function.^15^

Deciphering the structural basis for the diverse substrate specificities among the wide variety of type-III copper enzymes present in nature has long been a challenge. Within this report, we aim to investigate the enzymatic divergence within the AO subclass by characterizing the catalytic activity of *Sm*NspF and *Sg*GriF, to evaluate the potential of AOs to oxidize a broad range of small phenolic and *o*-aminophenolic compounds, as well as non-phenolic substrates such as aromatic amines and diamines. To achieve a comprehensive functional and structural understanding of this largely unstudied enzyme subclass, we further determine, for the first time, the three-dimensional structure of an AO, confirming the coupled binuclear copper site and elucidating the role of second sphere residues in comparison to bacterial and plant TYR crystal structures. These insights support progress in green biotechnology by promoting selective catalytic systems that avoid harsh oxidants and energy-intensive processes and utilize biocatalysts containing Earth-abundant metals rather than rare or precious ones.

## Results and discussion

### Expression and purification

To achieve maximum yields and purity of *Sm*NspF and *Sg*GriF, expression conditions were examined by adjusting the medium composition, specifically the NaCl and CuSO_4_ concentrations to support proper copper ion incorporation into the type-III copper center, as well as by optimizing the incubation temperature and incubation time (for detailed description of the protein expressions see SI Section S1.2). Heterologous expression using a *lac*-operon expression system and a GST-tag for affinity purification resulted in much higher expression efficiency for *Sm*NspF (yield ≈ 3.1 mg l^−1^ medium) compared to *Sg*GriF (yield ≈ 0.8 mg l^−1^ medium) (Table S2). *Sm*NspF also demonstrates higher purification efficiency than *Sg*GriF following the first affinity chromatography step (Table S2, Fig. S2). Additionally, *Sg*GriF requires an extra size-exclusion chromatography step (with a Superdex 75 10/300 GL; GE, Boston, MA, USA) to achieve purity levels higher than 95% (Table S2). The successful expression of purified *Sm*NspF and *Sg*GriF is verified by protein analysis with SDS-PAGE and intact protein mass spectrometry (SI Section S1.3, S1.4 and Fig. S4–S6). ESI-Orbitrap-MS revealed molecular masses of 35 837.41 ± 1.00 Da for *Sm*NspF and 35 736.16 ± 1.18 Da for *Sg*GriF, which matches well with the calculated mass for the recombinantly expressed enzymes (*Sm*NspF: 35 838.36 Da, *Sg*GriF: 35 737.28 Da). The successful incorporation of copper ions into the type-III copper center was confirmed by copper content analysis using 2,2′biquinoline, which revealed copper-to-enzyme ratios of 1.6 : 1 for *Sm*NspF and 1.5 : 1 for *Sg*GriF.^16^ UV-VIS spectroscopic investigation of *Sg*GriF after the addition of a 20-fold molar excess of hydroxylamine to an oxygen-saturated solution reveals the emergence of a band in the 325–355 nm range, indicating the formation of a metal–ligand charge transfer complex based on the *met* and/or *oxy* form (Fig. S7).^17,18^ The 280/345 nm absorption ratio of *Sg*GriF is approximately 0.11, which is comparable to the ratio observed for *Sm*NspF (approximately 0.12), as reported by Ginsbach *et al.* (2012), also following the addition of a 20-fold molar excess of hydroxylamine.^14^ Hydroxylamine reduces the resting *met*-form of the binuclear copper center, resulting in the formation of the *deoxy*-form, which enables dioxygen binding to generate the catalytically active *oxy*-form.^14^

### Kinetic investigation

**Fig. 1:**
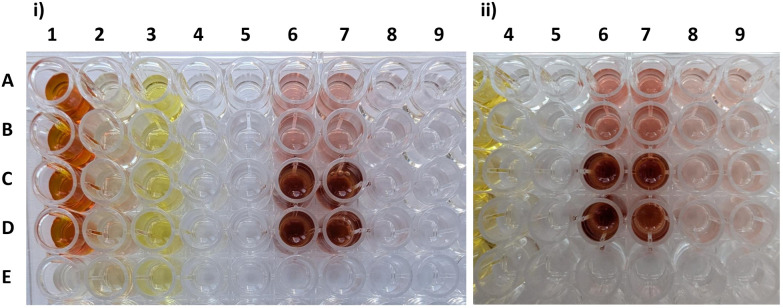
Row A and B: enzymatic reaction of 10 mM substrate respectively with *Sm*NspF, row C and D: enzymatic reaction of 10 mM substrate respectively with *Sg*GriF and row E: blank of the respective substrate (10 mM). (i) Enzymatic reaction of 10 mM substrate respectively with *Sm*NspF or *Sg*GriF after 10 min. Column 1: 3-amino-4-hydroxybenzoic acid, column 2: 4-amino-3-hydroxybenzoic acid, column 3: *o*-phenylendiamine, column 4: tyramine with 0.5 mM hydroxylamine, column 5: tyramine, column 6: 4-methylcatechol with 0.5 mM hydroxylamine, column 7: 4-methylcatechol, column 8: 4-methylphenol with 0.5 mM hydroxylamine, column 9: 4-methylphenol. (ii) Enzymatic reaction of 10 mM substrate respectively with *Sm*NspF or *Sg*GriF after 3.5 h. Column 5: tyramine, column 6: 4-methylcatechol with 0.5 mM hydroxylamine, column 7: 4-methylcatechol, column 8: 4-methylphenol with 0.5 mM hydroxylamine, column 9: 4-methylphenol. Substrate structures are presented in SI, Fig. S8–S11.

The monophenolase activity of *Sg*GriF was investigated by Suzuki *et al.* (2006)^19^ using l-tyrosine, 4-hydroxybenzaldehyde and phenol, with no activity observed. In our study, both enzymes show no visible monophenolase activity toward tyramine but exhibit slow enzymatic activity toward the monophenol 4-methylphenol (Fig. 1, columns 4, 5, 8 and 9).^6,14,19^ Notably, the addition of 0.5 mM hydroxylamine accelerated the oxidation of 4-methylphenol with both AOs, as indicated by a slight color change from colorless to pinkish observed within 3 min of reaction time (Fig. 1, *i* column 8). However, enzymatic oxidation of 4-methylphenol after three hours of reaction time was clearly observed with either *Sm*NspF or *Sg*GriF, both in the presence and absence of hydroxylamine. This suggests the existence of the resting as well as *deoxy*-form after this enzyme purification process, which includes the elution of GST-tagged enzymes with reduced glutathione during the first GST-tag affinity chromatography step (Fig. S2). The presence of the enzyme in its *deoxy*-form is essential for initiating the monophenolase reaction cycle of type-III copper enzymes.^20^

With regard to diphenolase activity, both the *met* and *oxy* forms can catalyze *o*-diphenol oxidation according to the reaction mechanism published in ref. 20. *Sm*NspF and *Sg*GriF show rapid diphenolase activity toward 4-methylcatechol (Fig. 1, columns 6 and 7; Table 1). Notably, *Sg*GriF displays higher catalytic efficiency for 4-methylcatechol compared to *Sm*NspF, indicated by a more intense color change and an approximately 2-fold higher *k*_cat_/*K*_m_ ratio (Fig. 1, columns 6 and 7; Table 1). Both AOs show diphenolase activity towards catechol, dopamine, 4-methylcatechol and 3,4-dihydroxybenzoic acid (3,4DHBA) (Fig. 1, columns 6 and 7; Table 1). However, no activity is observed with DOPAC.

**Table 1:** Enzyme kinetics of *Sm*NspF and *Sg*GriF

Substrate	AO	*K*_m_ [mM]	*k*_cat_ [s^−1^]	*k*_cat_/*K*_m_ [s^−1^ m M^−1^]
***o*-Diphenols (Fig. S9)**				
Benzene-1,2-diol (catechol)	*Sm*NspF	1.41 ± 0.20	1.275 ± 0.106	0.904 ± 0.149
	*Sg*GriF	2.58 ± 0.20	2.198 ± 0.107	0.852 ± 0.078
4-Methylbenzene-1,2-diol (4-methylcatechol)	***Sm*NspF**	**1.73 ± 0.35**	**5.648 ± 0.633**	**3.265 ± 0.755**
	***Sg*GriF**	**0.19 ± 0.01**	**1.140 ± 0.008**	**6.000 ± 0.319**
4-(2-Aminoethyl)benzol-1,2-diol (dopamine)	*Sm*NspF	10.04 ± 0.77	0.838 ± 0.048	0.083 ± 0.008
	*Sg*GriF	5.19 ± 0.17	1.054 ± 0.021	0.203 ± 0.008
3,4-Dihydroxybenzoic acid (3,4DHBA)	***Sm*NspF**	**4.65 ± 0.44**	**0.723 ± 0.056**	**0.155 ± 0.019**
	***Sg*GriF**	**5.47 ± 0.15**	**0.020 ± 0.001**	**0.004 ± 0.001**
(3,4-Dihydroxyphenyl)acetic acid (DOPAC)	*Sm*NspF	n. d.^b^	n. d.^b^	n. d.^b^
	*Sg*GriF	n. d.^b^	n. d.^b^	n. d.^b^
***o*-Aminophenols (**Fig. S10**)**^c^				
2-Aminophenol (2AP)	*Sm*NspF	9.35 ± 3.27	14.092 ± 4.011	1.507 ± 0.680
	*Sg*GriF	2.30 ± 0.06	26.481 ± 0.395	11.513 ± 0.346
2-Amino-4-methylphenol (2A4MP)	***Sm*NspF**	**6.13 ± 1.01**	**8.374 ± 0.103**	**1.366 ± 0.226**
	***Sg*GriF**	**0.50 ± 0.03**	**6.385 ± 0.172**	**12.770 ± 0.840**
3-Amino-4-hydroxybenzoic acid (3A4HBA)	*Sm*NspF	4.50 ± 0.83	12.086 ± 1.406	2.686 ± 0.981
	*Sg*GriF	n. d.^a^	n. d.^a^	n. d.^a^
4-Amino-3-hydroxybenzoic acid (4A3HBA)	***Sm*NspF**	**n. d.** b	**n. d.** b	**n. d.** b
	***Sg*GriF**	**n. d.** b	**n. d.** b	**n. d.** b
2-Amino-1,3-benzenediol (2-aminoresorcinol)	*Sm*NspF	12.14 ± 0.98	2.315 ± 0.166	0.19 ± 0.02
	*Sg*GriF	8.83 ± 1.68	10.402 ± 1.689	1.18 ± 0.30
**Aniline derivatives (**Fig. S11**)**				
Aniline	*Sm*NspF	n. d.^b^	n. d.^b^	n. d.^b^
	*Sg*GriF	n. d.^b^	n. d.^b^	n. d.^b^
1,2-Diaminobenzene (*o*-phenylenediamine)	***Sm*NspF**	**7.28 ± 0.24**	**0.018 ± 0.002**	**0.003 ± 0.001**
	***Sg*GriF**	**3.03 ± 0.22**	**0.007 ± 0.002**	**0.002 ± 0.001**

a Kinetic parameters could not be determined due to the low aqueous solubility of 3A4HBA (<9 mM), which prevented adequate definition of the Michaelis–Menten curve for *Sg*GriF.

b Kinetic values could not be determined due to insufficient or absent enzymatic activity.

c Kinetic parameters for *o*-aminophenol oxidation were determined by monitoring the formation of colored phenoxazinone oxidation products, reflecting the quinone imine-forming activity.

**Fig. 2:**
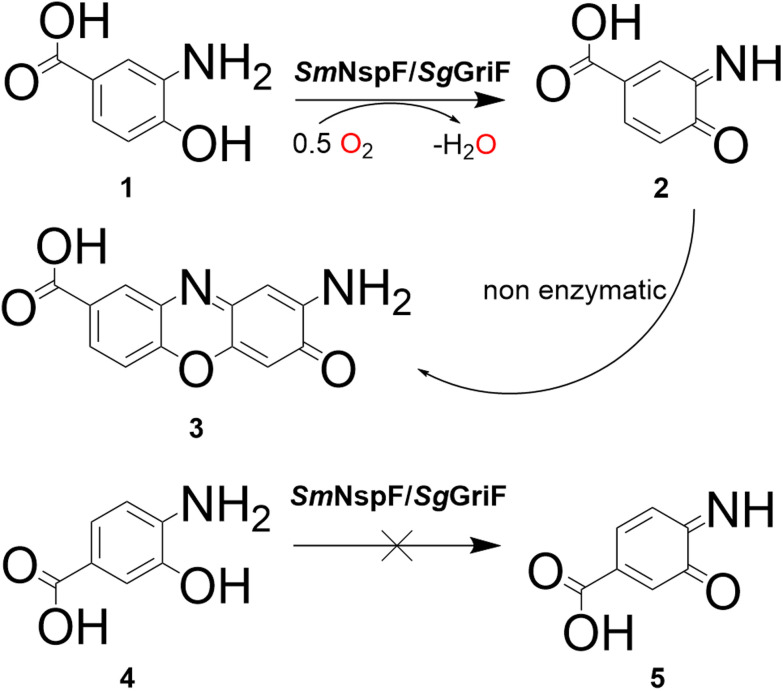
Quinone imine-forming activity of *Sm*NspF or *Sg*GriF, which shows their high substrate specificity for *para*-substituted *o*-aminophenols such as 3-amino-4-hydroxybenzoic acid (1) to form 2-aminophenoxazin-3-one-8-carboxylate (3). When the carboxylic group is *meta*-substituted, both enzymes do not accept 4-amino-3-hydroxybenzoic acid (4) as a preferred substrate for enzymatic oxidation to yield the quinone imine product (5).

*Sm*NspF and *Sg*GriF catalyze the oxidation of *o*-aminophenols to phenoxazinone products (quinone imine-forming activity) with substrates including 2-aminophenol (2AP), 2-amino-4-methylphenol (2A4MP), 3-amino-4-hydroxybenzoic acid (3A4HBA, 1) and 2-aminoresorcinol (Fig. 1 and 2, column 1; Table 1). Despite both enzymes showing enzymatic activity toward 3A4HBA (1), no activity was observed with 4-amino-3-hydroxybenzoic acid (4), which contains a carboxylic acid group in the *meta*-position relative to the phenolic hydroxy group (Fig. 1, columns 1 and 2; Fig. 2). These findings indicate that AOs display substrate specificity for *o*-aminophenols with functional groups at the *para*-position, which supports effective substrate binding at the active center.

Remarkably, AOs are accepting dihydroxyaniline substrates with two hydroxy groups at the *ortho*-position relative to the amino group as they exhibit enzymatic activity toward 2-aminoresorcinol (Table 1 and Fig. S10). The catalytic efficiency of this oxidation is much lower compared to 2-aminophenol which is demonstrated by an approximately 10-fold lower activity with *Sm*NspF and 4-fold lower activity with *Sg*GriF. Whereas tyrosinases demonstrate their ability to hydroxylate aniline derivatives, *Sm*NspF and S*g*GriF show no activity when incubated with aniline (Table 1).^21–23^ However, both AOs are capable of enzymatically oxidizing substrates beyond phenolic compounds, as they exhibit low activities toward *o*-phenylenediamine (Table 1 and Fig. 1, *i* column 3), similar to tyrosinases.^21–23^ This expands the known enzymatic versatility of AOs, demonstrating their ability to catalyze the oxidation of aromatic *o*-diamines for the first time. The oxidation of *o*-phenylenediamine with AOs proceeds more slowly compared to catechol (over 300-fold lower) and 2AP (over 600-fold lower). An explanation for the considerable difference between the substrate classes (aromatic *o*-diamines *vs. o*-diphenols *vs. o*-aminophenols) is their relative tendency to undergo deprotonation, given the significantly higher p*K*_a_ of anilines (p*K*_a_ ∼ 25) compared to phenols (p*K*_a_ ∼ 10) and the amino group in aminophenols (p*K*_a_ ∼ 5).^22,24^ Hence, substrate deprotonation is a pivotal and rate-determining step in the enzymatic reaction mechanism.^22^

Differences in enzymatic kinetics between *Sm*NspF and *Sg*GriF are most evident in their substrate preferences for *para*-substituted *o*-diphenols and *o*-aminophenols. Thus, *Sm*NspF displays strikingly higher activity toward substrates with a carboxylic acid group (approximately 38-fold higher for 3,4DHBA), whereas *Sg*GriF exhibits greater activity toward those substituted with a methyl group in the *para*-position (approximately 2-fold higher for 4-methylcatechol and 9-fold for 2A4MP) (Table 1). The exceptional preference of *Sm*NspF for *o*-aminophenol substrates bearing a carboxylic acid group is evident not only in comparison to *Sg*GriF but also to tyrosinases.^6,21–23^ Based solely on the observed reaction rates, a clear distinction emerges between AOs and tyrosinases. In contrast to kinetic data collected from two fungal tyrosinases (*Neurospora crassa* and *Agaricus* bisporus), which exhibit a strong preference for *o*-diphenolic substrates over their corresponding *o*-aminophenol analogs, both AOs examined in this study show predominantly higher or comparable catalytic efficiencies with *o*-aminophenol substrates (Table 1, Fig. S9–S10).^21–23^ For example, *Sg*GriF oxidizes 2-aminophenol approximately 5-fold faster than catechol and *Sm*NspF oxidizes 3A4HBA approximately 2-fold faster than the corresponding *o*-diphenol 3,4DHBA (Table 1). Enzymatic activities of mushroom tyrosinase published by Muñoz-Muñoz *et al.* (2011) show a 70-fold higher catalytic efficiency for catechol and 29-fold higher for 3,4DHBA oxidation in comparison to their *o*-aminophenol analogs.^21^

### Structural evaluation

**Fig. 3:**
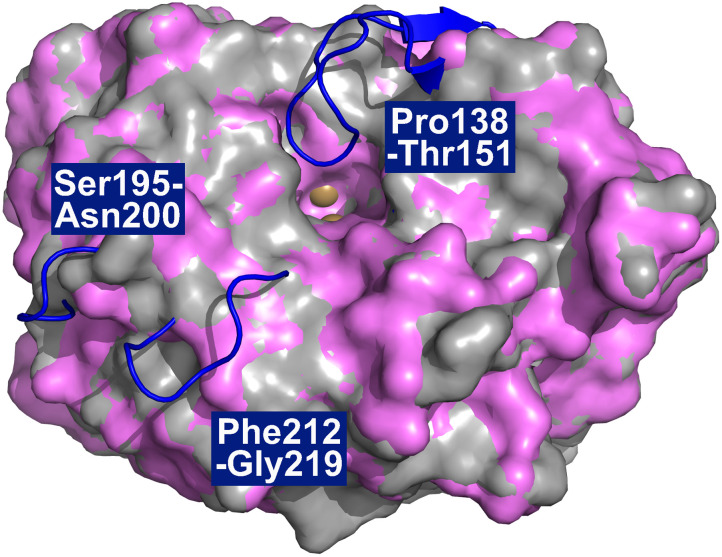
Structure alignment of *Sm*NspF (PDB ID: 9T62), *Sc*TYR (PDB ID: 1WX2) and *Sa*TYR (PDB ID: 6J2U). The copper center is depicted as brown spheres. The grey surface (*Sa*TYR) and violet surface (*Sc*TYR) show a high degree of structural overlap between the two tyrosinases. The loops in blue highlight the three elongated regions in *Sm*NspF (PL1: Pro138-Thr151, PL2: Ser195-Asn200 and PL3: Phe212-Gly219).

Protein crystals of *Sm*NspF were obtained from a buffered solution at pH 7.5 containing 20 mM TRIS, 8% PEG 8000 and 800 mM Li_2_SO_4_ as the precipitating agent (SI Section S1.8). An *in crystallo* activity using 2-amino-4-methylphenol confirmed that the crystallized protein remained active, as evidenced by the rapid and intense coloration of the protein crystal (Fig. S15). The crystal structure of *Sm*NspF represents the first experimental structural determination of an AO (PDB ID: 9T62). The *Sm*NspF core architecture closely resembles previously reported polyphenol oxidase (PPO) structures.^7,20^ A great structural similarity is observed with the crystal structure of the tyrosinase from *Streptomyces castaneoglobisporus* (*Sc*TYR, PDB ID:1WX2, Uniprot ID: Q83WS2), sharing 48.17% sequence identity and *Streptomyces avermitillis* (*Sa*TYR, PDB ID: 6J2U, Uniprot ID: Q79Z38), sharing 43.17% sequence identity.^13,25^ A notable distinction between *Sm*NspF and bacterial tyrosinases is the presence of three elongated loop regions, conserved among AOs and they are not present in tyrosinases (Fig. 3). One of these loops (Peptide Loop 1 (PL1)) lies near the active site and includes a 14-residue insertion in both *Sm*NspF (Pro138–Thr151) and *Sg*GriF (Pro138–Thr151) compared to the structure of *Sc*TYR and *Sa*TYR (Fig. 3).^14^ PL1 (Pro138–Thr151) and Peptide Loop 3 (PL3: Phe212–Gly219) could possibly restrict the active site and subsequently play a role in substrate recognition due to their proximity to the binding pocket (distance to CuA of PL1 ≈ 9.5 Å and PL3 ≈ 15.5 Å) (Fig. 3). In contrast, Peptide Loop 2 (PL2: Ser195–Asn200) exhibits a much larger distance from the active-site pocket (distance to CuA of PL3 ≈ 19.5 Å) (Fig. 3). Yet the function of this conserved AO loop in enzyme activity is still unclear. The monomeric *Sm*NspF is ellipsoidal, measuring approximately 48 × 46 × 32 Å. The secondary structure is predominantly α-helical, with the enzyme core formed by a four α-helix bundle (α2, α3, α5, α6). Each of the two catalytic copper ions is coordinated by three histidines from the α-helices. CuA is ligated by His39, His58, and His67, with His39 positioned mid-helix on α2. CuB is coordinated by His222, His226, and His248, located in the middle of α6 and α7. The presence of an ‘oxygen moiety’ in the electron density map between the copper ions suggests that the enzyme was crystallized in its met-form. The dicopper center features a Cu–Cu distance of 3.8 Å, comparable to that of the met-form of *Sc*TYR (3.7 Å).^18^

**Fig. 4:**
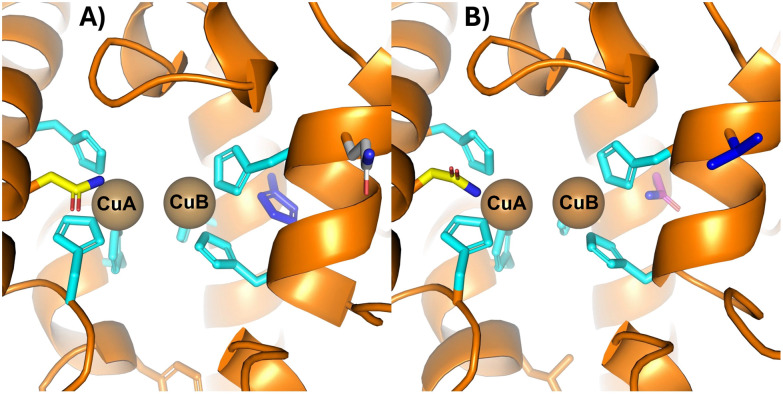
Structure alignment of *Sm*NspF (PDB ID: 9T62) and *Sg*GriF (AlphaFoldDB model B1VTI5) with PyMOL Molecular Graphics System, Version 3.0 Schrödinger, LLC.^29,30^ (A) Active center of *Sm*NspF with coordinating histidines in cyan and the activity selector Asn43 in yellow. The seventh histidine is depicted in blue, and the second activity controller (His_B2+1_) Gln227 is depicted in grey. Copper ions are depicted as brown spheres. (B) Active center of *Sg*GriF with coordinating histidines in cyan and the activity selector Asn43 in yellow. *Sg*GriF harbors Asn247, depicted in magenta, instead of the seventh histidine, and the second activity controller (His_B2+1_) Val227 is shown in blue. Copper ions are depicted as brown spheres. The structure alignment of *Sm*NspF (PDB ID: 9T62) and *Sm*NspF AlphaFold model (AlphaFoldDB model D6RTB9) is shown in Fig. S16.

Multiple structure to function relationships in polyphenol oxidases (PPOs) have been reported in earlier studies and comprehensively reviewed by Kampatsikas and Rompel (2021).^20^ Second-shell residues at the active-site play a crucial role in substrate specificity and reaction rates, despite only minor residue differences among PPOs.^26,27^ For example, the residue positioned after the second Cu_B_-coordinating histidine, known as the second activity controller, alters the local electrostatic environment (Fig. 4).^20^ In tyrosinases *To*TYR-02 and *Jr*TYR, this position contains a small hydrophobic residue (Ile in *To*TYR-02 and Leu in *Jr*TYR), whereas in *To*TYR-06, and in catechol oxidases *Ib*CO, and *Vv*CO, it is occupied by a long, positively charged residue (Arg or Lys) capable of stabilizing acidic groups on substrate's tail, as the His_B2+1_ residue interacts directly with the substrate's tail.^20,27,28^ A positively charged amino acid at this position such as Arg enhances the active site's affinity for negatively charged substrates, whereas the opposite effect occurs for positively charged substrates.^28^ The substrate-guiding effect of the second activity controller (His_B2+1_) can also help to explain the functional differences between *Sm*NspF and *Sg*GriF, given the high sequence homology between tyrosinases and AOs (Fig. 4).

**Fig. 5:**
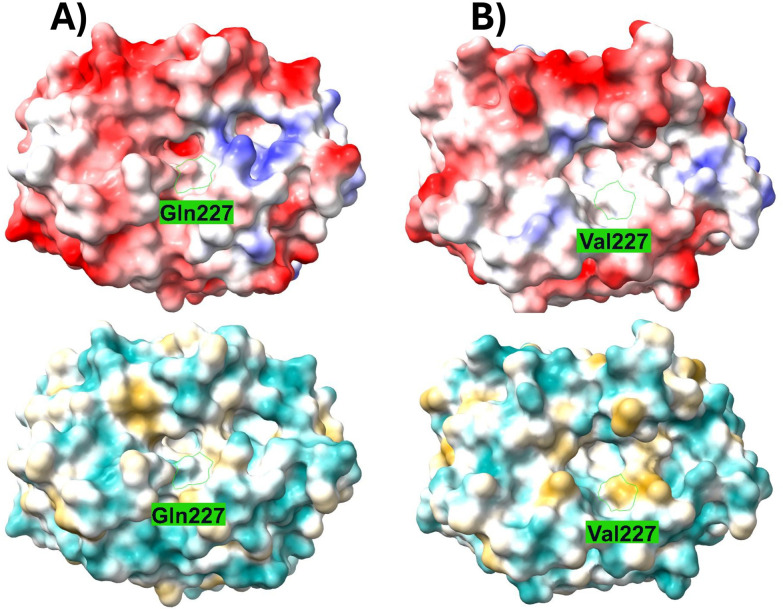
Electrostatic and hydrophobic surface map of (A) *Sm*NspF (PDB ID: 9T62) and (B) *Sg*GriF (AlphaFoldDB model B1VTI5) with ChimeraX 1.11.^29,30,34^ Residues Gln227 (*Sm*NspF) and Val227 (*Sg*GriF) are highlighted in green. The negative potential surface is depicted in red, and the positive potential surface is depicted in blue. The hydrophilicity is depicted in cyan, and hydrophobicity is depicted in yellow. The calculated binding-pocket volume with a maximum depth of 7.2 Å is 218 Å^3^ in *Sm*NspF and 230 Å^3^ in *Sg*GriF.

In *Sm*NspF, His_B2+1_ is a polar Gln, which can act as a hydrogen-bond donor, potentially explaining its preference for substrates with carboxyl groups that serve as hydrogen-bond acceptors.^31^ In contrast, *Sg*GriF contains a hydrophobic Val at this position, resulting in a significantly lower reaction rate toward carboxylic-tail substrates such as 3,4-dihydroxybenzoic acid and 3-amino-4-hydroxybenzoic acid. This divergence between the two AOs can be visualized in the electrostatic and hydrophobic surface maps (Fig. 5). On the one hand, residue Gln227 in *Sm*NspF increases the electrostatic potential in the outer binding-pocket region in comparison to the surface map of *Sg*GriF with Val227. On the other hand, Val227 in *Sg*GriF significantly increases local hydrophobicity, which hinders polar interactions with substrates harboring a carboxylate tail. Please note that PPOs with a seventh histidine residue, such as *Sm*NspF, exhibit high affinity for substrates with a carboxylic tail, whereas those containing leucine at this position show greater affinity for substrates with a decarboxylated tail.^20,32,33^ The binding-pocket volumes of both enzymes calculated with a maximum depth of 7.2 Å are very similar (approximately 218 Å^3^ in *Sm*NspF and 230 Å^3^ in *Sg*GriF). One possible cause of the slightly tighter binding pocket of *Sm*NspF is the different orientation of Peptide Loop 1, which in *Sm*NspF shows a greater interaction with the outer binding-pocket region (Fig. 3 and 5). Despite the divergent orientation of Peptide Loop 1, both enzymes exhibit nitroso-forming activity, indicating that this additional loop is likely not pivotal for this activity. The slightly smaller binding pocket in *Sm*NspF compared to *Sg*GriF is consistent with the observed kinetic values. On the one hand, *Sm*NspF exhibits relatively high substrate specificity for 3A4HBA due to its tightened binding pocket; on the other hand, it shows much lower reaction rates (approximately tenfold lower) for the other investigated *o*-aminophenols compared to the larger binding pocket in *Sg*GriF.

*Sg*GriF lacks the seventh histidine and instead contains an asparagine in the homologous position (Fig. 4). In tyrosinases, the presence of amino acids Asp or Asn at the positions of the first (His_B1+1_) and second (His_B2+1_) activity controller residues creates an environment that enhances the basicity of the conserved His_B1_ and His_B2_ residues, enabling them to act as proton acceptors.^20^ This structural feature is an indicator of monophenolase activity, based on previous mutagenesis studies focusing on the first (His_B1+1_) and second (His_B2+1_) activity controllers.^20^ The thereby activated His_B1_ and His_B2_ can deprotonate monophenolic substrates, facilitating the monophenolase function.^20^ In *Sm*NspF and *Sg*GriF, the His_B1+1_ position is occupied by a conserved Gly residue, while the His_B2+1_ position contains a polar Gln in *Sm*NspF and a nonpolar Val in *Sg*GriF (Fig. 4). Despite this structural difference from tyrosinases, both enzymes display slow monophenolase activity toward 4-methylphenol (Fig. 1, *ii* columns 8 and 9).^12^ A hypothesis for the weak monophenolase activity of both enzymes is that the relatively flexible His_A2_, in the absence of the thioether bridge, can move closer to the copper center. The thioether bridge is present in most structurally characterized plant and fungal PPOs.^20^ Without the thioether bond, as in *Sm*NspF and *Sg*GriF as well as in most bacterial tyrosinases, His_A2_ could possibly interact with the acidic residue (water keeper Glu207), increasing its basicity enough to deprotonate monophenolic substrates (Fig. 1, columns 8 and 9).^20,35^ Recent experimental investigations combined with quantum mechanics/molecular mechanics studies by Kipouros *et al.* (2022) show an alternative mechanism, highlighting that the monophenolic hydrogen reacts directly with the μ:η^2^:η^2^-peroxide dicopper(ii) center which acts as a proton acceptor.^36–38^

## Conclusion

The investigation of the enzymatic activities of *Sm*NspF and *Sg*GriF, which belong to a highly versatile subclass of the binuclear copper protein family, confirmed their overall similarity in substrate specificity, while also revealing differences in catalytic efficiencies between them. This study provides new insight into the AOs by presenting crystal structure information about the overall structure and dicopper center of *Sm*NspF and by elucidating how small sequence variations in the second coordination sphere can influence the distinct substrate preferences of *Sm*NspF and *Sg*GriF. The substrate-guiding effect of the second activity controller (His_B2+1_) in *Sm*NspF and *Sg*GriF determines the binding affinity for carboxyl-containing substrates, thereby leading to increased or decreased reaction rates with compounds such as 3,4DHBA and 3A4HBA. Regarding monophenolase activity, *Sm*NspF and *Sg*GriF were found to be active without hydroxylamine, while the addition of hydroxylamine further accelerated this activity. Beyond the classical mono-, *o*-diphenolic, and *o*-aminophenolic substrates, both AOs are also capable of oxidizing 2-aminoresorcinol and *o*-phenylenediamine, albeit at markedly slower rates. This observation not only extends the known substrate spectrum of AOs, but also suggests a previously underappreciated flexibility in their enzymatic activities. Since aniline derivatives are widely used in dyes, pharmaceuticals, and synthetic polymers, exploiting AO reactivity toward *o*-phenylenediamines offers opportunities in the synthesis of functional polymers, sensors, and novel building blocks.^39–42^ Resorcinol derivatives are applied in pharmaceuticals, tire manufacturing, hair dyes, and dermatology, but pose serious toxicological risks as it can disrupt the central nervous system, impair red blood cell function, and act as an endocrine disruptor.^43–50^ They represent major phenolic wastewater contaminants from industries such as paper, resin, textile, plastic, coking, tanning, rubber, pharmaceuticals, herbicides, fungicides, and petroleum production.^46^ The ability of AOs to oxidize such pollutants underscores their potential for sustainable bioremediation, which utilizes biocatalysts containing Earth-abundant metals rather than rare or precious ones. Overall, these findings highlight the ability of AOs to oxidize a great variety of small compounds and provide the opportunity for rational engineering of catalytic properties from sequence-level information to enable reactions previously considered inaccessible for biocatalysts.

## Author contributions

Hoa Le Xuan: conceptualization, data curation, formal analysis, investigation, methodology, project administration, validation, visualization, and writing – original draft. Annette Rompel: conceptualization, funding acquisition, project administration, resources, supervision, and writing – original draft. All the authors have approved the final manuscript.

## Conflicts of interest

There are no conflicts to declare.

## Supplementary Material

QI-013-D5QI02495A-s001

## Data Availability

The data that support the findings of this study are openly available in phaidra at https://phaidra.univie.ac.at/o:2178036. Crystallographic dataset are openly available at European Synchrotron Radiation Facility DOI: https://doi.org/10.15151/ESRF-DC-2333941181. Ref. 51–65 are cited in the supplementary information (SI). Supplementary information containing detailed experimental procedures on heterologous expression, analytical characterization, kinetic investigation, and X-ray data collection is available. See DOI: https://doi.org/10.1039/d5qi02495a.
