# Supplementary material for: Structural insights into ortho-aminophenol oxidases: kinetic and crystallographic characterization of SmNspF and SgGriF
Source: Inorg Chem Front. 2026 Jan 30;13(9):3786–94. doi: 10.1039/d5qi02495a (PMC12982950; doi:10.1039/d5qi02495a)
Supplement: QI-013-D5QI02495A-s001 [file QI-013-D5QI02495A-s001.pdf]

Supplementary Information (SI) for Inorganic Chemistry Frontiers.
This journal is © the Partner Organisations 2026

Supporting Information

Structural Insights into *ortho*-Aminophenol Oxidase: Kinetic and Crystallographic Characterization of *Sm*NspF and *Sg*GriF

Hoa Le Xuan[a,b] *and Annette Rompel**[a,b]

[a] H. Le Xuan, M.Sc., A. Rompel
Universität Wien, Fakultät für Chemie,
Institut für Biophysikalische Chemie,
Josef-Holaubek Platz 2, 1090 Vienna (Austria),
E-mail: annette.rompel@univie.ac.at
Homepage: https://www.bpc.univie.ac.at

[b] H. Le Xuan, M.Sc., A. Rompel
Vienna Doctoral School in Chemistry (DoSChem),
Universität Wien,
Währinger Straße 42, 1090 Vienna (Austria)
Homepage: https://doschem.univie.ac.at/

Table of Contents

List of abbreviations 4

1. Experimental Procedures 6

    1.1 Cloning of *Sm*NspF 6

    1.2 Heterologous Expression in *E. coli* and Purification of *Sm*NspF 6

    1.3 SDS-PAGE Analysis 7

    1.4 Intact Protein Mass Spectrometry 7

    1.5 Cloning and Heterologous Expression of *Sg*GriF 8

    1.6 Copper Quantification with 2,2´-Biquinoline 8

    1.7 Substrate Activity Assays 8

    1.8 Crystallization, Data collection and Processing of *Sm*NspF 9

2. Results 10

  2.1. Supplementary Figures 10

    Figure S1: Enzymatic Activity of AO/TYR/CO 10

    Figure S2: First Affinity Chromatography of *Sm*NspF 11

    Figure S3: Second Affinity Chromatography of *Sm*NspF 11

    Figure S4: SDS-PAGE 12

    Figure S5: ESI-Orbitrap Mass Spectrum of *Sm*NspF in Positive Mode 12

    Figure S6: ESI-Orbitrap Mass Spectrum of *Sg*GriF in Positive Mode 13

    Figure S7: UV-VIS Spectrum of *Sg*GriF 13

    Figure S8: Structures of Monophenolic Substrates 14

    Figure S9: Structures of *o*-Diphenolic Substrates 14

    Figure S10: Structures of *o*-Aminophenol Substrates 15

    Figure S11: Structure of an *o*-Diaminobenzene 15

    Figure S12: Enzyme Kinetics with *o*-Diphenols 16

    Figure S13: Enzyme Kinetics with *o*-Aminophenols 17

    Figure S14: Enzyme Kinetics with *o*-Phenylendiamine 18

    Figure S16: Building Asn43 Side Chain in Crystal Structure of *Sm*NspF 19

  2.2 Supplementary Tables 19

    Table S1: Designed Primers with Annealing Temperatures. 19

    Table S2: Purification of Expressed Enzymes 20

    Table S3 Measurement Wavelength and Extinction Coefficient of the Substrate Activity Assays 20

    Table S4: Data Collection and Processing 21

3. References 22

List of abbreviations

**×g**	times gravity
**2A4MP**	2-amino-4-methylphenol
**2AP**	2-aminophenol
**3A4HBA**	3-amino-4-hydroxybenzoic acid
**3A4HBAL**	3-amino-4-hydroxybenzaldehyde
**3A4HBAm**	3-amino-4-hydroxybenzamide
**3A4HBS**	3-amino-4-hydroxybenzenesulfonic acid
**AC**	affinity chromatography
**AO**	*o*-aminophenol oxidase
**ATCC**	American Type Culture Collection
**ATP**	adenosine triphosphate
**CO**	catechol oxidase
**DE3**	λDE3 lysogen (prophage carrying the T7 RNA polymerase gene under control of the lacUV5 promoter)
**DNA**	deoxyribonucleic acid
**E**	elution step
E. coli	*Escherichia coli*
**EDTA**	ethylenediaminetetraacetic acid
**ESI**	Electrospray ionization
**ESRF**	European Synchrotron Radiation Facility
**FIP**	Fédération Internationale de Pharmacie
**FT**	flow through
**fwd**	forward
**GE**	General Electric
**Gln**	glutamine
**GSH**	reduced glutathione
**GST**	glutathione S-transferase
**GSTrap FF**	Glutathione Sepharose Fast Flow column
**h**	hours
**HRV3C**	human rhinovirus (serotype 14B) protease 3C
lac	Lactose
**Leu**	leucine
**Ile**	isoleucine
**IPTG**	isopropyl β-D-1-thiogalactopyranoside
**LB**	lysogeny broth: 10 gl^{-1} peptone or tryptone, 5 gl^{-1} yeast extract and 10 gl^{-1} NaCl in water
**LTQ**	linear ion trap Q
**M**	molar; mole per liter; in pseudomolecular ions: mass
**MS**	mass spectrometry
**n. d.**	not determined
**NIH**	National Institute of Health
**NL**	normalization level
**mAU**	milli absorption units; 1 mAU corresponds to an absorbance of 0.001 cm^{-1}
**NEB**	New England Biolabs
**OD_{600}**	optical density at a wavelength of 600 nm
**PAGE**	polyacrylamid gel electrophoresis
**PES**	Polyethersulfone
**PCR**	polymerase chain reaction
**RT**	room temperature
**rev**	reverse
**rpm**	revolutions per minute

**S/N**	signal-to-noise ratio
**SDS**	sodium dodecyl sulfate; sodium lauryl sulfate
**SI**	supporting information
***Sm*NspF**	*o*-aminophenol oxidase originating from *Streptomyces murayamaensis*
**Spec. Act.**	specific activity
**T**	temperature
**TRIS**	tris(hydroxymethyl)aminomethane; 2-amino-2-(hydroxymethyl)propane-1,3-diol
**TYR**	tyrosinase (EC 1.14.18.1)
**Tyr**	tyrosine
**U**	unit (of enzymatic activity); 1 U = 1 µmolmin^{-1} of transformed substrate (2A4MP)
**USA**	United States of America
**Val**	valine
**(v/v)**	volume per volume
**W**	washing step
**(w/v)**	weight per volume

1. Experimental Procedures

1.1 Cloning of SmNspF. The *Sm*NspF gene was amplified from *Streptomyces murayamaensis* (resource identifier #21414, ATCC, Manassas, Virginia, USA) using PCR with Q5 High-Fidelity DNA Polymerase (NEB, Ipswich, Massachusetts, USA), following the manufacturer's instructions and employing the primers *Sm*NspF-FWD and *Sm*NspF-REV (**Table S1**). The resulting PCR product was inserted into the pGEX-6P-SG vector using Esp3I restriction sites (NEB; recognition sequence: 5´…CGTCTC(N)1|…3´, 3´…GCAGAG(N)5|…5´) and T4 DNA ligase (NEB) in a one-step reaction at 30 °C for 90 minutes, in accordance with the supplier's protocol. The recombinant plasmid was sequenced by Microsynth Austria GmbH, Vienna, Austria to confirm correct gene insertion. Verified constructs were then introduced into *E. coli* BL21 (DE3) cells using heat shock transformation.

1.2 Heterologous Expression in E. coli and Purification of SmNspF. The enzyme *Sm*NspF was fused at its N-terminus to a Glutathione-S-Transferase (GST) tag using the pGEX-6P vector, which includes separate *tac* promoters and a *lac* operator for expression control. A human rhinovirus 3C protease (HRV3C) cleavage site (LEVLFQ|GP) is included between the GST-tag and *Sm*NspF to enable enzymatic proteolysis with HRV3C. Expression was performed in auto-induction medium at pH 7 containing 5.0 g/L yeast extract (Merck, Darmstadt, Deutschland), 10.0 g/L tryptone (Carl Roth, Karlsruhe, Germany), 2.0 mM $MgSO_4$, 500.0 mM NaCl, 25.0 mM Na_2HPO_4, 25.0 mM KH_2PO_4, 50.0 mM NH_4Cl, 5.0 mM Na_2SO_4, 5.0 g/L glycerol, 0.5 g/L D-glucose, and 2.0 g/L α-D-lactose, supplemented with 100 µg/mL ampicillin. Saturated overnight cultures of *E. coli* BL21 (DE3) harboring the expression vector were diluted into fresh auto-induction medium in baffled Erlenmeyer flasks to an initial OD_{600} of 0.02 and incubated at 37 °C with shaking at 230 rpm until the OD_{600} reached 0.50–0.60. Following this, $CuSO_4$ was added to a final concentration of 2.0 mM, and incubation was continued for 24 hours at 15 °C with shaking at 230 rpm. Cells were harvested by centrifugation at 6000×g for 20 minutes at 4 °C. After discarding the supernatant, cell pellets were resuspended in lysis buffer (20 mM sodium phosphate, 300 mM NaCl, 1 mM EDTA, pH 7.4) containing 0.5 g/L lysozyme (≥45000 FIP U/mg, Carl Roth, Karlsruhe, Germany), 1 mM phenylmethylsulfonyl fluoride, and 1 mM benzamidine. The suspension was incubated at 4 °C for 45 minutes, followed by five freeze-thaw cycles using liquid nitrogen and a 25 °C water bath. Afterward, 2 mM $MgCl_2$ and 0.02 g/L DNase I (VWR International, Vienna, Austria) were added and the mixtures were incubated at 25 °C for 15 minutes at 200 rpm. The lysate was then centrifuged at 8000×g for 60 minutes at 4 °C, and following this the supernatant was filtered using bottle top filter (PES, 45 µm, Thermo Fisher, Massachusetts, USA).

Protein purification was conducted at 4 °C using an ÄKTA Purifier (GE Healthcare, Solingen, Germany). The first step involved affinity chromatography (**Table S2**) on a 5 mL GSTrap FF column (GE) with a binding buffer containing 50 mM TRIS-HCl and 200 mM NaCl (pH 8.0). The column was washed with

60 mL of washing buffer (20 mM TRIS, 300 mM KCl, 10 mM $MgCl_2$, 5 mM ATP, pH 8.0) and bound proteins (including target enzyme) were eluted with binding buffer supplemented with 15 mM reduced glutathione (**Fig. S2**). The buffer exchange of eluted protein fraction was performed by using a dialysis tubing (Dialysis membrane Membra-Cel, 34 mm, Carl Roth, Karlsruhe, Germany). The sample was loaded into the dialysis tubing and dialyzed against the binding buffer four times for 2 hour each at 4 °C to remove glutathione. To cleave the GST tag, the fusion protein was incubated overnight at 4 °C with in-house produced GST-tagged HRV3C protease at a 1:50 mass ratio (protease:fusion protein).[1] A second affinity chromatography purification step (**Table S2**) using the same GSTrap FF column (GE) enabled the separation of the cleaved target protein, which did not bind to the column, from the GST-tagged protease and free GST tag, both of which remained bound (**Fig. S3**). The target protein was then concentrated using a Vivaspin ultrafiltration device (Sartorius, Vienna, Austria) with a 30 kDa molecular weight cut-off membrane, exchanged into binding buffer (50 mM TRIS-HCl, 200 mM NaCl, pH 8.1), and stored at 4 °C. Protein concentration was determined spectrophotometrically at 280 nm according to Lambert-Beer law using the extinction coefficient of *Sm*NspF (82,850 $M^{-1}cm^{-1}$, all cysteine residues are reduced) calculated via ExPASy ProtParam.[2, 3] The protein sequence has been taken from NIH genetic sequence database (GenBank: BAJ08174.1).

***1.3 SDS-PAGE Analysis*.** Electrophoresis was performed using a Mini-PROTEAN Tetra Cell System (Bio-Rad, Vienna, Austria). The purity of the recombinantly expressed *Sm*NspF was evaluated using SDS-PAGE with 13% acrylamide gels (**Fig. S4**). Protein samples were mixed with 6×loading buffer (375 mM TRIS-HCl, pH 6.8, 9% (w/v) SDS, 50% (w/v) glycerol, 0.03% (w/v) bromophenol blue, and 9% (v/v) β-mercaptoethanol), then heated at 99 °C for 10 minutes. A molecular weight standard (Precision Plus Protein Dual Color Standard, Bio-Rad) was included for reference. Gels were stained overnight with a Coomassie solution (0.02% Coomassie Brilliant Blue G-250, 5% (w/v) aluminium sulfate, 10% (v/v) ethanol, and 2 g/L o-phosphoric acid), then washed with a solution of 10% (v/v) ethanol and 20 g/L *o*-phosphoric acid.

***1.4 Intact Protein Mass Spectrometry*.** Purified proteins were analyzed via intact mass spectrometry using an UltiMate 3000 Nano LC system coupled to either an LTQ-Orbitrap Velos (Thermo Fisher Scientific, Bremen, Germany) equipped with a nanospray ionization source (**Fig. S5-S6**) at the Mass Spectrometry Center, University of Vienna. The data was recorded in positive ion mode. Instrument calibration was performed using the ESI-L Low Concentration Tuning Mix (Agilent, Part No. G1969-85000). The *Sm*NspF solution was buffer exchanged to 5 mM ammonium acetate (pH 7.4) using a Vivaspin 500 ultrafiltration device with a 30 kDa molecular weight cutoff (Sartorius). The resulting sample was diluted to 1 µM in an aqueous solution containing 2% (v/v) acetonitrile and 1% (v/v) formic acid prior to MS analysis. The ion transfer capillary was maintained at 300 °C with an electrospray

voltage of 2.1 kV. Samples were first loaded onto a trap column, followed by separation on an Accucore C4 analytical column (15 cm × 75 µm, 2.6 µm particle size, 150 Å pore size, Thermo Fisher) at a flow rate of 300 nL/min. The mobile phase consisted of solvent A (98% water, 2% acetonitrile, 0.1% formic acid) and solvent B (80% acetonitrile, 20% water, 0.1% formic acid).

***1.5 Cloning and Heterologous Expression of SgGriF*.** The cloning, heterologous expression of *Sg*GriF and purification (**Table S2**) was carried out according to Le Xuan *et al.* 2025.[4]

***1.6 Copper Quantification with 2,2´-Biquinoline*.** An enzyme sample containing 600 µg in 0.1 M sodium phosphate buffer (pH 6.0) was mixed with 50 µL of ascorbic acid solution to denature the protein and fully reduce the copper ions to Cu(1+). The mixture was then adjusted to a final volume of 400 µL using the same sodium phosphate buffer (0.1 M, pH 6.0). Subsequently, 600 µl of 2,2´-biquinoline solution (0.5 mg/ml in glacial acetic acid) was added, and the mixture was incubated at room temperature for 10 minutes to allow formation of the copper(I)-2,2´-biquinoline complex. The concentration of this complex was determined spectrophotometrically at 546 nm using the Lambert-Beer law and an extinction coefficient of 6300 $M^{-1}cm^{-1}$.[2, 5] Absorbance was measured with a Shimadzu UV-1800 spectrophotometer (Shimadzu, Kyoto, Japan), using a blank solution containing 350 µl sodium phosphate buffer (0.1 M, pH 6.0), 50 µL of ascorbic acid solution and 600 µl 2,2´-biquinoline (0.5 mg/ml in glacial acetic acid).

***1.7 Substrate Activity Assays*.** Enzymatic activities were assayed at RT using a TECAN infinite M200 reader (Tecan, Salzburg, Austria) in a 96-well microplate with a total reaction volume of 200 µl. The assays were carried out with 5 µg/ml enzyme (in 50 mM TRIS-HCl, 200 mM NaCl, pH 7.5) and respective substrate with eight different concentrations. All tests were carried out in triplicates at room temperature, monitoring the formation of oxidation products (**Table S3)**. The final kinetic constant values were obtained through nonlinear regression, applying the Levenberg–Marquardt algorithm with either Equation A (no noticeable substrate inhibition) or Equation B (marked substrate inhibition).[6] In these regressions (**Fig. S11-13**), the weighting factor for each data point was calculated as the ratio of the mean specific activity, so that its contribution to the total sum of squared residuals reflected its influence relative to the current parameter estimates of the model. Substrate activity assays for the enzymatic oxidation of *o*-aminophenol were performed by monitoring the formation of colored phenoxazinone oxidation products, reflecting quinone imine–forming activity.

Equation A: Michaelis-Menten kinetics.

$$v = \frac{V_{max} \cdot [S]}{K_m + [S]}$$

v: rate of product formation

V_{max}: maximal rate of product formation under substrate saturation conditions

[S]: substrate concentration

K_M: Michaelis constant, substrate concentration at which v = 0.5 of V_{max}

<u>**Equation B**: Michaelis-Menten kinetics with substrate inhibition.</u>

$$v = \frac{V_{max} \cdot [S]}{K_M + [S](1 + \frac{[S]}{K_{I,S}})}$$

$K_{I,S}$: substrate inhibition constant

v: rate of product formation

V_{max}: maximal rate of product formation under conditions of substrate saturation

[S]: substrate concentration

K_M: Michaelis constant, substrate concentration at which v = 0.5 of V_{max}

***1.8 Crystallization, Data collection and Processing of SmNspF*.**

Crystallization was carried out using the hanging-drop vapor-diffusion method in 15-well EasyXtal plates (Qiagen, Venlo, Netherlands). Crystals were grown at 4 °C by mixing 5 µL of *Sm*NspF solution (10 mg mL^{-1}) with 4 µL of reservoir solution (20.0 mM Tris, 16.1 mM HCl, 8% PEG 8000, and 800 mM Li_2SO_4, pH 7.5). Crystals typically appeared after 7 days. *Sm*NspF crystals were mounted on nylon loops and flash-cooled in liquid nitrogen after brief soaking in a cryoprotectant solution containing 20 mM Tris, 8% PEG 8000, 800 mM Li_2SO_4, and 20% glycerol. Diffraction data for wild-type *Sm*NspF were collected at 100 K on beamline ID30A-3 at the ESRF (Grenoble, France). Spot finding, indexing, parameter refinement, and integration were performed with Global Phasing AutoProc and STARANISO.[7, 8] Initial phases for the enzyme structure, were obtained by molecular replacement with MOLREP, followed by structure determination in CCP4 Cloud.[9, 10] The structural model was manually rebuilt in COOT (version 0.9.8.93) and iteratively refined in CCP4 Cloud with Refmac5.[10, 11, 12] This process was repeated until no further improvements could be achieved. The final models were validated using the MolProbity server before deposition in the Protein Data Bank (apo-SlPPO1: PDB 6HQJ; holo-SlPPO1: PDB 6HQI).[13] Data collection and processing statistics are summarized in **Table S4**, confirming the high quality of the datasets. Figures were generated using PyMOL Molecular Graphics System, Version 3.1.6.1 Schrödinger, LLC. Coordinates and structure factors were deposited in the PDB under the accession code 9T62.

2. Results

2.1. Supplementary Figures

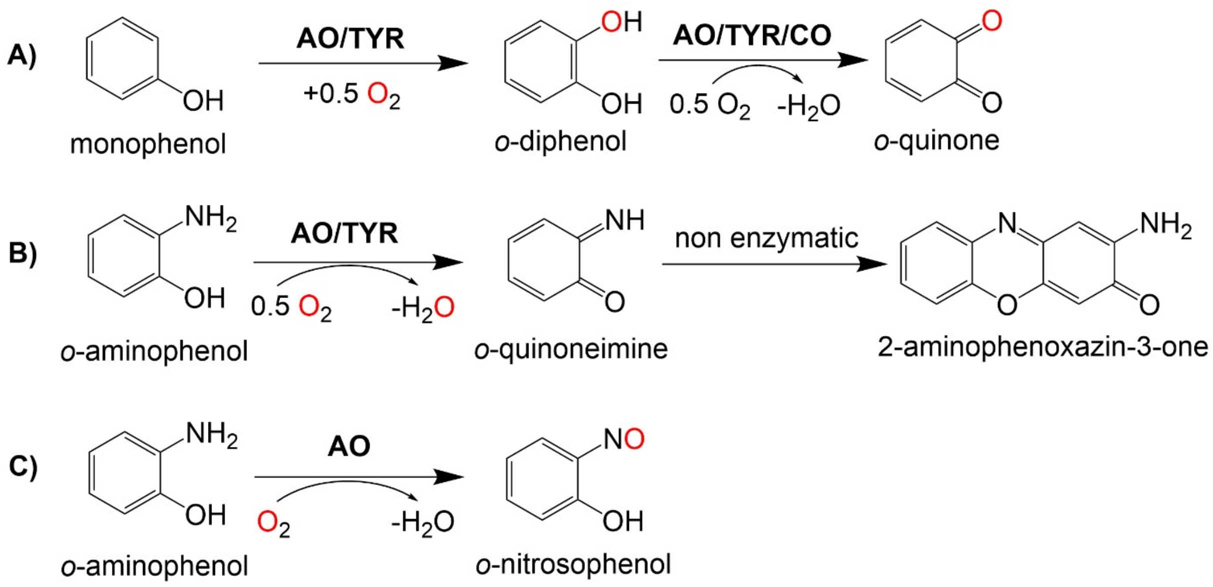

Figure S1: Enzymatic Activity of AO/TYR/CO. **A**) Monophenolase activity of phenol oxidation to *o*-diphenol catalyzed by tyrosine (TYR) or *o*-aminophenol oxidase (AO) and diphenolase activity of *o*-diphenol to *o*-quinone by TYR AO or catechol oxidase (CO). **B**) Quinone imine-forming activity of *o*-aminophenol oxidation to *o*-quinone imine catalyzed by AO or TYR and subsequent non enzymatic reaction to 2-aminophenoxazin-3-one.[14] **C**) Nitroso-forming activity of *o*-aminophenol oxidation to *o*-nitrosophenol catalyzed by AO.[15]

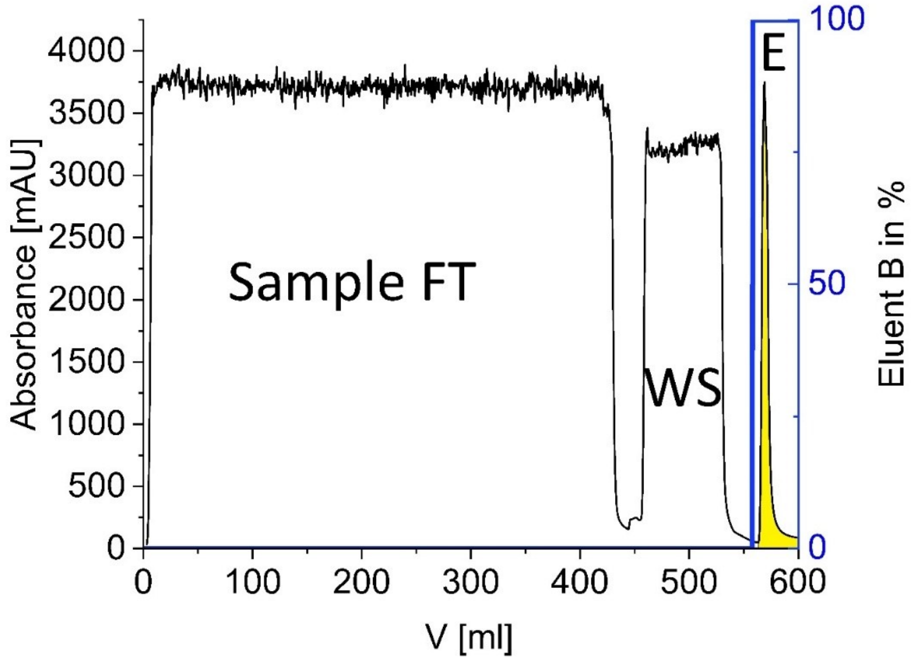

***Figure S2: First Affinity Chromatography of SmNspF*:** Affinity chromatography with GSTrap FF (GE) of *Sm*NspF GST-Tag fusion protein (FT (Flow Through), WS (Washing Step), E (Elution Step)) was conducted at flow rate 1 ml/min and measured at 280 nm.

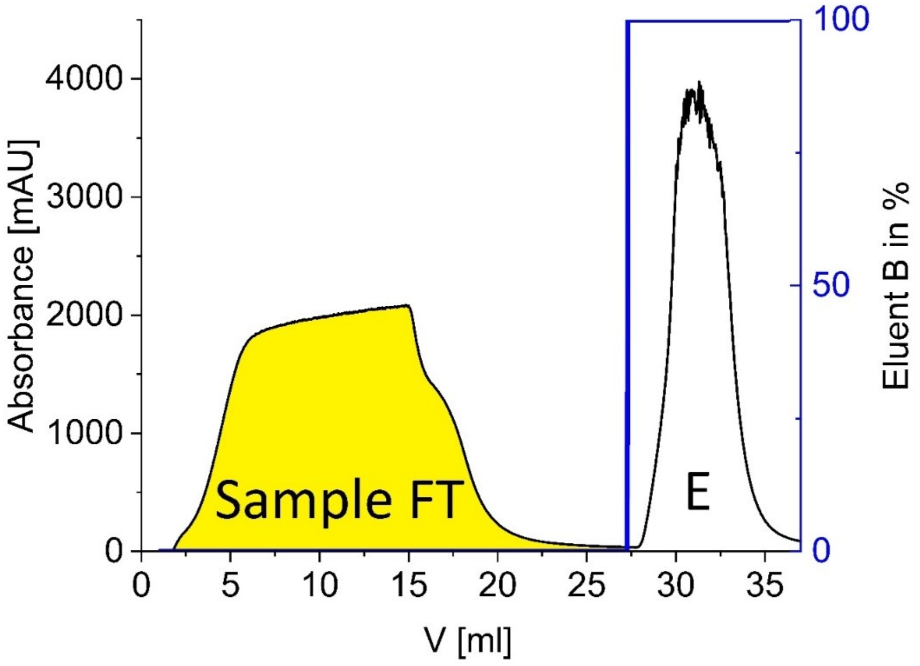

***Figure S3: Second Affinity Chromatography of SmNspF*:** Affinity chromatography after GST-cleavage with GSTrap FF (GE) of *Sm*NspF (FT (Flow Through) and E (Elution Step)) was conducted at flow rate 1 ml/min and measured at 280 nm.

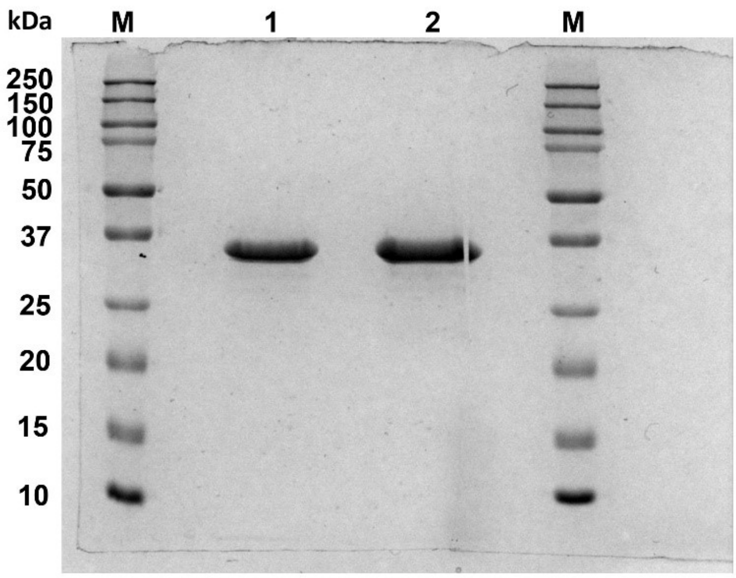

Figure S4: SDS-PAGE. Reduced and untruncated 13% acrylamide SDS PAGE. Lane 1: 10 µg *Sm*NspF, lane 2: 10 µg *Sg*GriF and the lanes M: molecular weight marker (Precision Plus Protein Dual Color Standards (Bio-Rad Laboratories GmbH, Feldkirchen, Germany)); the size of the standard bands is given in kDa. The samples in lanes 1 and 2 indicate successful purification of *Sm*NspF (theoretical mass: 35,838.36 Da) and *Sg*GriF (theoretical mass: 35,737.28 Da).

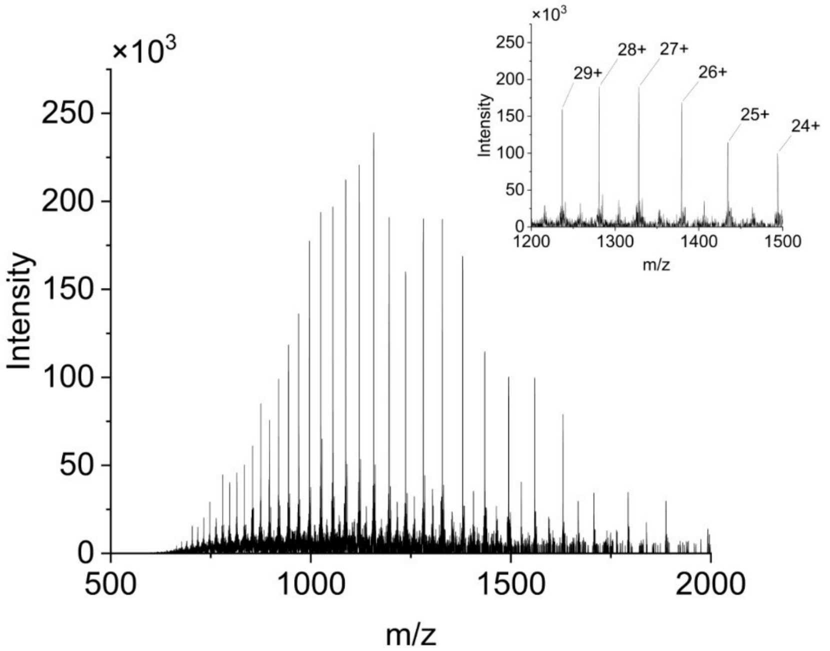

Figure S5: ESI-Orbitrap Mass Spectrum of SmNspF in Positive Mode. To the left the entire mass spectrum of *Sm*NspF is depicted, on the upper right a zoom-in to the most intense signals originating from the pseudomolecular ions $[M + 35H^+]^{29+}$ to $[M + 30H^+]^{24+}$ is shown. Theoretical mass of *Sm*NspF: 35,838.36 Da; experimental mass of *Sm*NspF: 35,837.41±1.00 Da S/N:258, NL:1.82E+006.

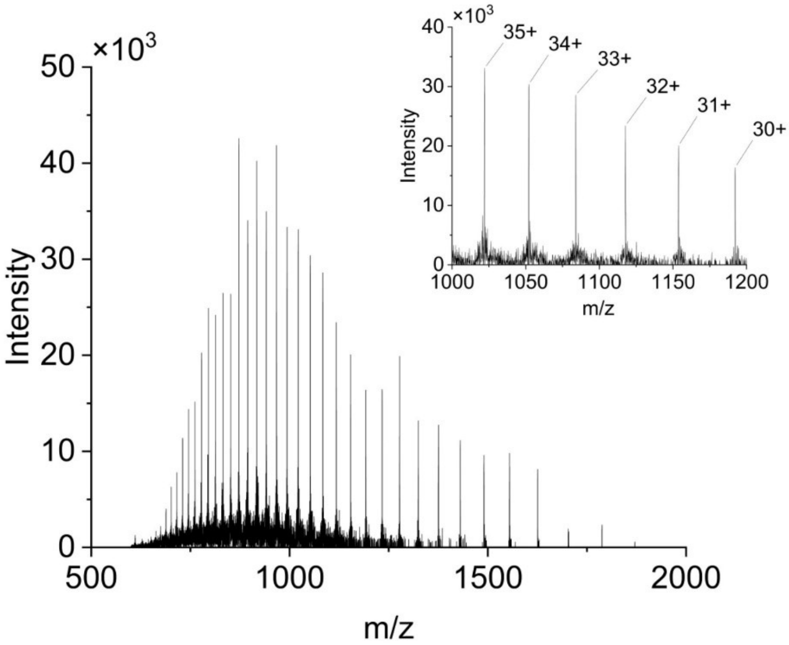

Figure S6: ESI-Orbitrap Mass Spectrum of SgGriF in Positive Mode. To the left the entire mass spectrum of *Sg*GriF is depicted, on the upper right a zoom-in to the most intense signals originating from the pseudomolecular ions $[M + 35H^{+}]^{35+}$ to $[M + 30H^{+}]^{30+}$ is shown. Theoretical mass of *Sg*GriF: 35,737.28 Da; experimental mass of *Sg*GriF: 35,736.16±1.18 Da S/N:218, NL:6.10E+005.

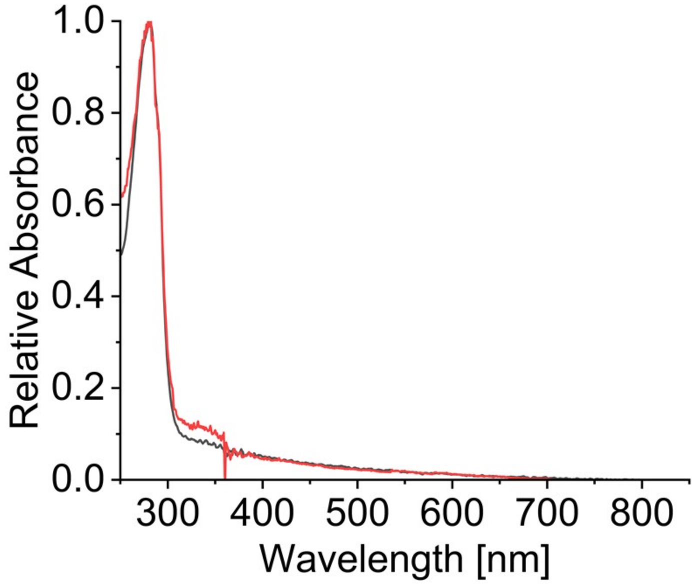

Figure S7: UV-VIS Spectrum of SgGriF. Black curve: *Sg*GriF without hydroxylamine; red curve: *Sg*GriF after addition of 20 eq. of hydroxylamine and gentle stirring for 5 min. The light source change of the Shimadzu UV-1800 spectrophotometer was at 360 nm.

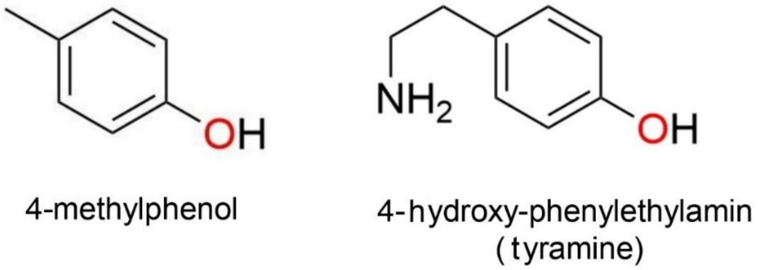

Figure S8: Structures of Monophenolic Substrates.

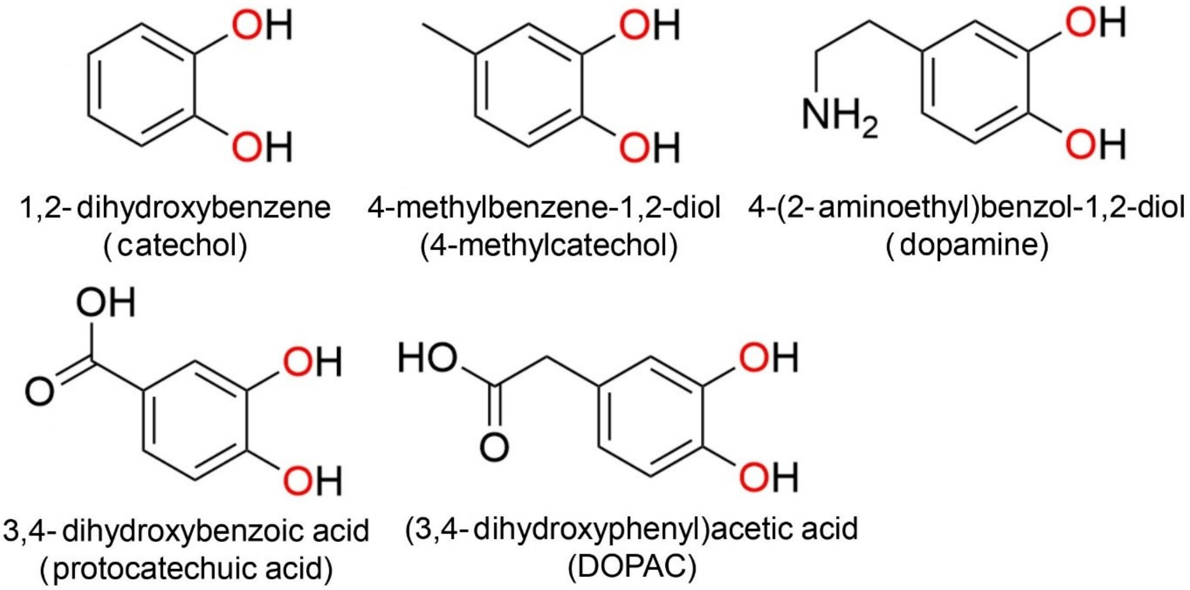

Figure S9: Structures of o-Diphenolic Substrates.

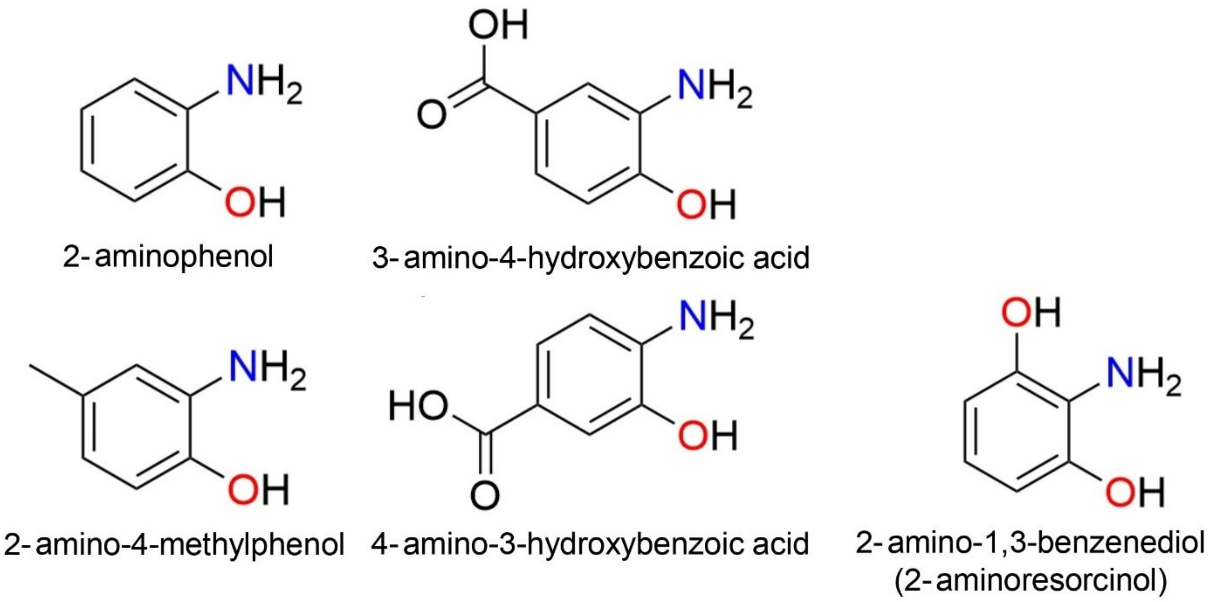

Figure S10: Structures of o-Aminophenol Substrates.

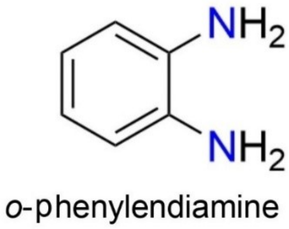

Figure S11: Structure of an o-Diaminobenzene.

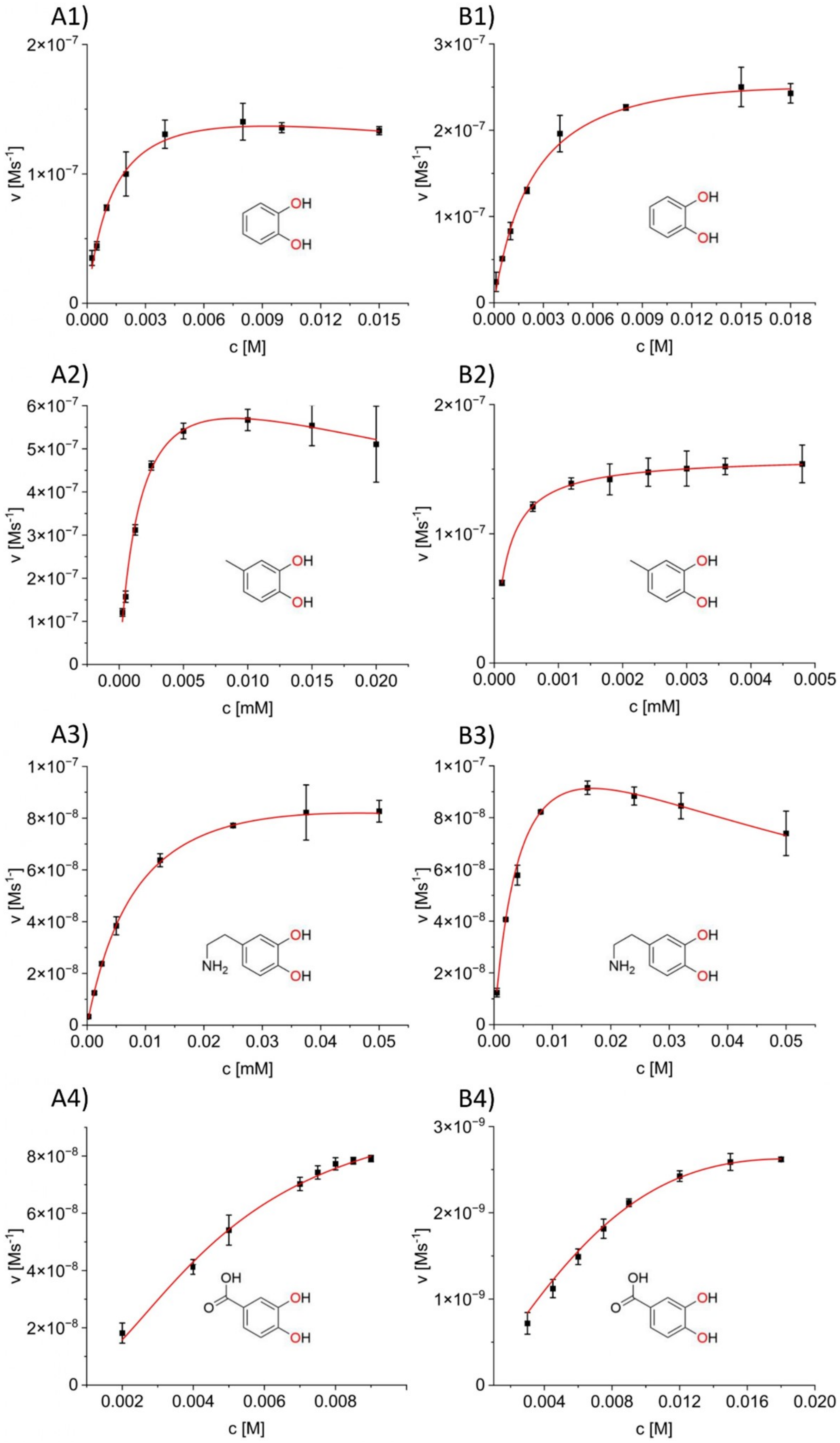

Figure S12: Enzyme Kinetics with o-Diphenols. A1-A4: Michaelis-Menten curves with *Sm*NspF. B1-B4: Michaelis-Menten curves with *Sg*GriF. K_m and K_{cat} are shown in Table 1.

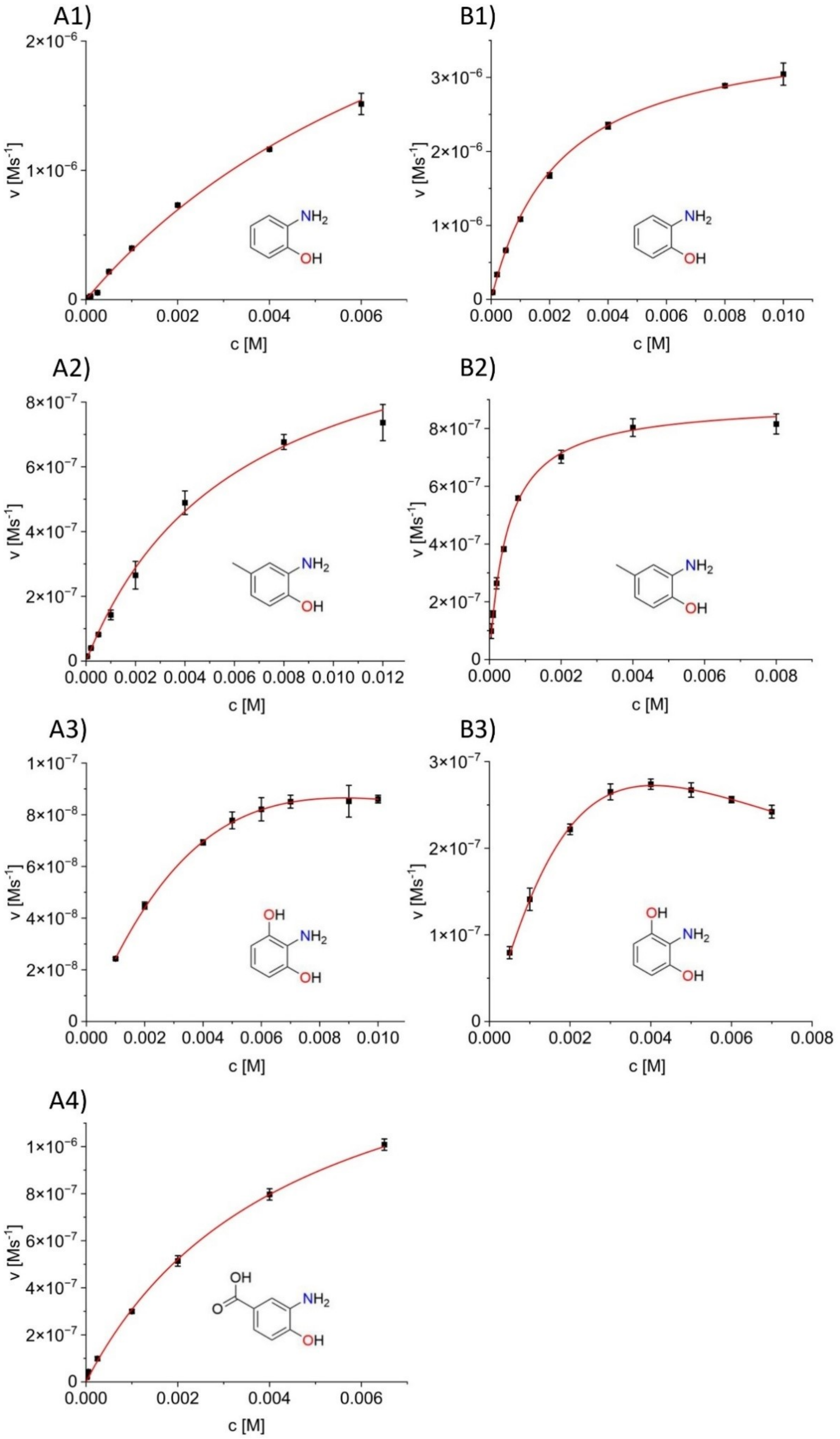

Figure S13: Enzyme Kinetics with o-Aminophenols. A1-A4: Michaelis-Menten curves with *Sm*NspF. B1-B3: Michaelis-Menten curves with *Sg*GriF. K_m and K_{cat} are shown in Table 1.

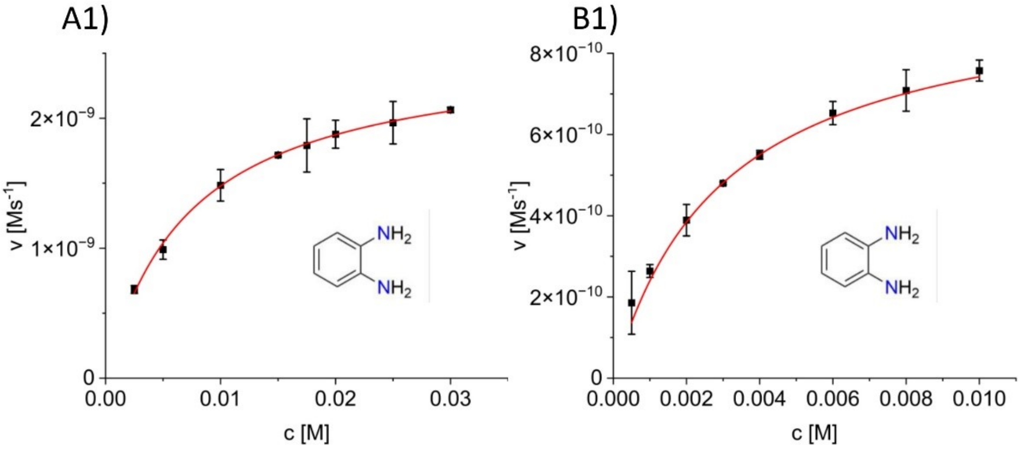

Figure S14: Enzyme Kinetics with o-Phenylendiamine. A1: Michaelis-Menten curves with *Sm*NspF. B1: Michaelis-Menten curves with *Sg*GriF. K_m and K_{cat} values are shown in Table 1.

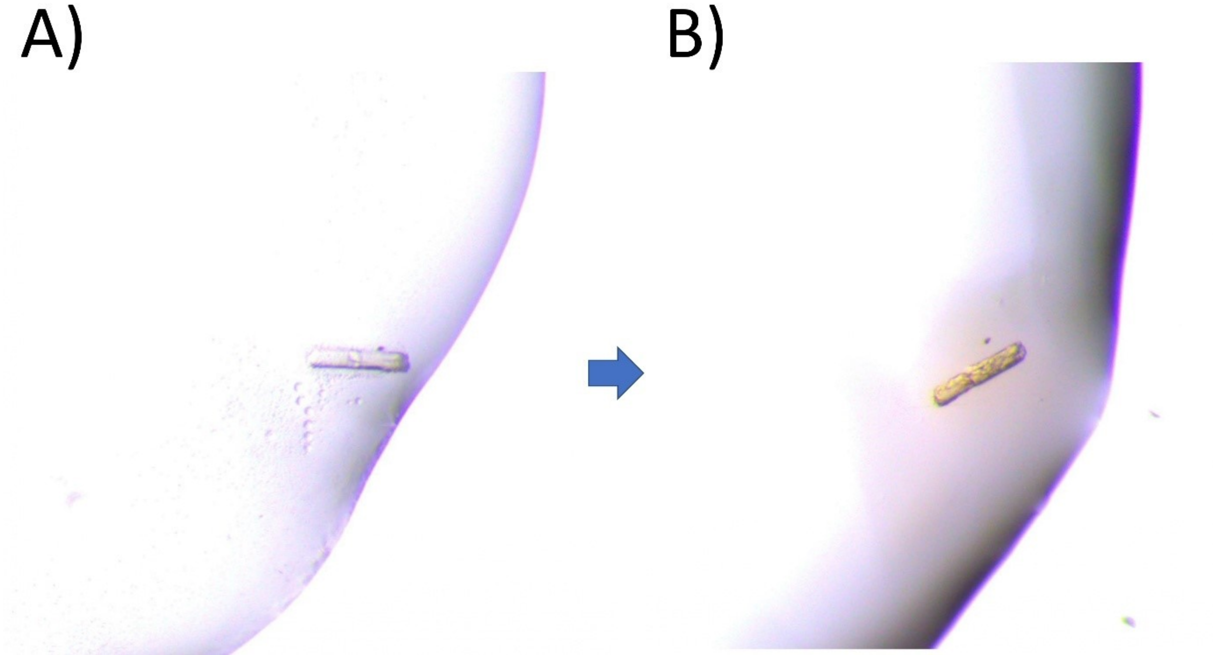

Figure S15: In Crystallo Activity of SmNspF Protein Crystal. A: *Sm*NspF crystal added to fresh reservoir solution (20 mM Tris, 8% PEG 8000, and 800 mM Li_2SO_4, pH 7.5). B: Addition of 2A4MP (10 mM final concentration) to the *Sm*NspF crystal in fresh reservoir solution resulted in substrate oxidation, visible as a yellow color change, accompanied by slow crystal decomposition.

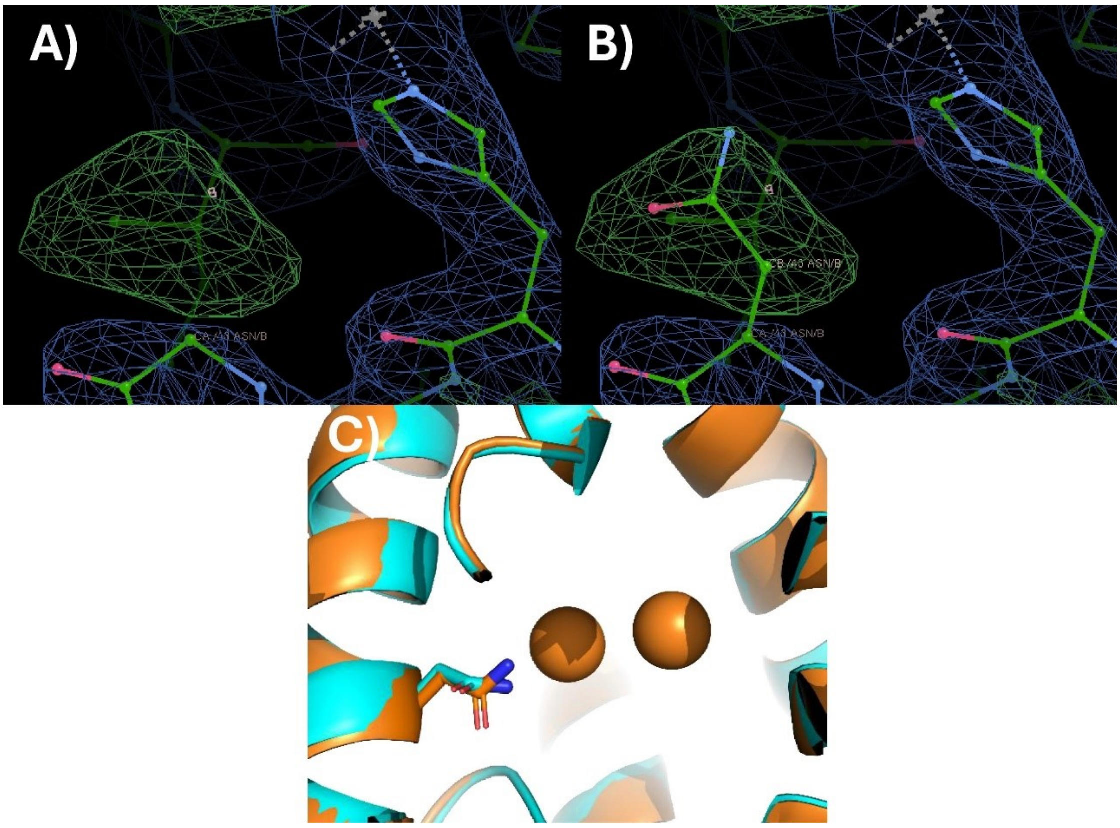

Figure S16: Building Asn43 Side Chain in Crystal Structure of SmNspF. A: Omit map of Asn43 without side chain. B: Omit map of Asn43 with modelled side chain. Copper atoms in omit maps are depicted as grey spheres. C: Structure alignment of *Sm*NspF crystal structure (depicted in orange) and SmNspF AlphaFold model (AlphaFoldDB model D6RTB9, depicted in cyan) with PyMOL Molecular Graphics System, Version 3.0 Schrödinger, LLC.[16, 17] Both copper atoms are shown as brown spheres. Comparison of the crystal structure with the AlphaFold model of *Sm*NspF reveals a small discrepancy in the side-chain orientation of Asn43. The observed orientation of the Asn43 side chain is compatible with the experimentally observed electron density. It is also possible for the side chain to be flipped by 180°, such that the carbonyl group is directed toward the dicopper center. The side chain within the binding pocket is relatively dynamic and likely samples multiple conformations.

2.2 Supplementary Tables

Table S1: Designed Primers with Annealing Temperatures.

Name	Sequence	Annealing T
*Sm*NspF-FWD	5′ agctcgtctccaATGACGCACGTCCGCAG 3′	69 °C
*Sm*NspF-REV	5′ agctcgtctcatcccTCAGGCGTACGTGTAGAACCG 3′	69 °C

Note: Non-pairing bases are written in small letters.

Table S2: Purification of Expressed Enzymes.

enzyme	purification step	spec. activity[a] [Umg^{-1}]	total activity [U]	yield [%]
***Sm*NspF**	crude lysate	0.0017 ± 0.0001	148	100
	GST-tagged enzyme after first AC	0.6818 ± 0.0450	100	68
	GST-cleaved enzyme after second AC	5.8278 ± 0.4697	68	46
***Sg*GriF**[b]	crude lysate	0.0064 ± 0.0003	101	100
	GST-tagged enzyme after AC	1.4702 ± 0.1302	38	38
	GST-cleaved enzyme after AC	5.2140 ± 0.4581	13	13
	purified enzyme after SEC	7.8138 ± 0.1705	6	6

[a] Specific activities were determined by oxidation of 10 mM 2A4MP with 5 µg/ml of enzyme at 400 nm and the corresponding first standard deviations are given after the ± sign. [b] The data shown are taken from Le Xuan *et al.* (2025).[4]

Table S3 Measurement Wavelength and Extinction Coefficient of the Substrate Activity Assays.

Substrate	(nm)	(M^{-1}cm^{-1})
1,2-diaminobenzene (o-phenylenediamine)	420	16700[18, 19]
2-Aminophenol (2AP)	433	2280[a]
3,4-dihydroxybenzoic acid (3,4DHBA)	398	2129[20]
2-amino-1,3-benzenediol (2-Aminorecorcinol)	400	1469[a]
2-amino-4-methylphenol (2A4MP)	400	3621[4]
3-Amino-4-hydroxybenzoic acid	433	1440[a]

4-Methylcatechol	400	1400[21]
Catechol	410	1623[22]
Dopamine	480	3300[22]

[a] In a total volume of 200 µL, the substrates at concentrations of 0.05, 0.10, 0.50, and 1.00 mM were oxidized in Tris–HCl buffer (50 mM Tris, 200 mM NaCl, pH 7.5) containing 6.25 mM $NaIO_4$, and absorption values were recorded at room temperature in a 96-well microplate using a TECAN Infinite M200 reader (Tecan, Salzburg, Austria). Extinction coefficients were subsequently determined spectrophotometrically according to the Beer–Lambert law by calculating a linear regression.[3]

***Table S4: Data Collection and Processing*.**

**DATA COLLECTION**	
Diffraction source	ID30A-3, ESRF
Temperature [K]	100 K
Detector	Eiger 4M (DECTRIS)
Diffraction source	ID30A-3, ESRF
Wavelength	0.968
Space group	P 3
Cell dimensions	a=86.19, b=86.19, c=88.25,<br>α=90.00, β=90.00, γ=120.00
Resolution	74.64 - 1.95 (2.00-1.95)
Total reflections	163690 (7631)
Unique reflections	52735 (3556)
Multiplicity	3.10 (2.10)
Completeness	99.10 (95.10)
I / σI	2.40 (0.00)
Wilson B-factor	46.29
Rmerge	0.43 (-34.02)
Rmeas	0.52 (-45.40)
Rpim	0.30 (-29.73)
CC½	0.92 (0.06)
**MODEL PROPERTIES**	
Number of models	1
Number of chains	2
Overall number of atoms (non-H)	5010
in macromolecules	4979
in ligands	4 CU / 2 CL
in solvent	23
Overall number of H-D atoms	0
**REFINEMENT**	
Resolution	74.64 - 2.40
Reflections in refinement	28394
Reflections in free set	1416
Rwork	0.179
Rfree	0.237

FSC average	0.987
RMSD bonds	0.0096
RMSD angles	1.968
Ramachandran favoured (%)	91.1
Ramachandran allowed (%)	8.9
Ramachandran outliers (%)	0.0
Rotamer outliers	2.2
Clash score	4.1
MolProbity score	1.98
Average B-factor	61.0
for macromolecules	61.1
for ligands	70.4 CU / 55.8 CL
for solvent	40.5
PDB ID	9T62

3. References

[1] M. Pretzler, A. Rompel, Tyrosinases: a family of copper-containing metalloenzymes, *ChemTexts*, 2024, **10**, 12–59. doi: 10.1007/s40828-024-00195-y.

[2] E. Gasteiger, C. Hoogland, A. Gattiker, S. Duvaud, M. Wilkins, R. Appel, A. Bairoch, in Proteomics Protocols Handbook, J. Walker, *Humana Press*, Totowa 2005, The Use of Resorcinol in Rubber Compositions, 571–607. doi: 10.1385/1-59259-890-0:571

[3] D. F. Swinehart, The Beer-Lambert Law, *J. Chem. Educ.*, 1962, **39**, 333. doi: 10.1021/ed039p333

[4] H. Le Xuan, F. Panis, A. Rompel, Identification of an Activity Selector for the Nitroso-Forming Activity in Bacterial Type-III Copper Enzymes, *Angew. Chem., Int. Ed.*, 2025, **64**, e202501560. doi: 10.1002/anie.202501560.

[5] P. M. Hanna, R. Tamilarasan, D. R. McMillin, Cu(I) analysis of blue copper proteins, *Biochem. J.*, 1988, **256**, 1001-1004. doi: 10.1042/bj2561001.

[6] D. Marquardt, An Algorithm for Least-Squares Estimation of Nonlinear Parameters, *J. Soc. Ind. Appl. Math.*, 1963, *11*, 431–441. doi: 10.1137/0111030. doi: 10.1137/0111030.

[7] C. Vonrhein, C. Flensburg, P. Keller, A. Sharff, O. Smart, W. Paciorek, T. Womack, G. Bricogne, Data processing and analysis with the autoPROC toolbox, *Acta Crystallogr.*, 2011, **D67**, 293-302. doi: 10.1107/S0907444911007773.

[8] I. J. Tickle, C. Flensburg, P. Keller, W. Paciorek, A. Sharff, C. Vonrhein, G. Bricogne, STARANISO, United Kingdom: Global Phasing Ltd, Cambridge, 2016. http://staraniso.globalphasing.org/cgi-bin/staraniso.cgi.

[9] A. Vagin, A. Teplyakov, MOLREP: an Automated Program for Molecular Replacement, A. *J. Appl. Cryst.*, 1997, **30**, 1022-1025. doi: 10.1107/S0021889897006766.

[10] E. Krissinel, A. A. Lebedev, V. Uski, C. Ballard, R. M. Keegan, O., Kovalevskiy, R. A. Nicholls, N. S. Pannu, P. Skubak, J. Berrisford, M. Fando, B., Lohkamp, M. Wojdyr, A. J. Simpkin, J. M. H. Thomas, C. Oliver, C., Vonrhein, G. Chojnowski, A. Basle, A. Purkiss, M. N. Isupov, S. McNicholas, E. Lowe, J. Trivino, K. Cowtan, J. Agirre, D. J. Rigden, I. Uson, V. Lamzin, I. Tews, G. Bricogne, A. G. W. Leslie, D. Brown, CCP4 Cloud for structure determination and project management in macromolecular crystallography, *Acta Crystallogr.*, 2022, **D78**, 1079-1089. doi: 10.1107/S2059798322007987.

[11] P. Emsley, K Cowtan, Coot: model-building tools for molecular graphics, *Acta Cryst.*, 2004, **D60**, 2126-2132. doi: 10.1107/S0907444904019158.

[12] G. N. Murshudov, A. A. Vagin, E. J. Dodson, Refinement of macromolecular structures by the maximum-likelihood method, *Acta Crystallogr.*, 1997, **D53**, 240-255. doi: 10.1107/S0907444996012255.

[13] V. B. Chen, W. B. Arendall III, J. J. Headd, D. A. Keedy, R. M. Immormino, G. J. Kapral, L. W. Murray, J. S. Richardson, D. C. Richardson, MolProbity: all-atom structure validation for macromolecular crystallography, *Acta Crystallogr.*, 2010, **D66**, 12-21. doi: 10.1107/S0907444909042073.

[14] H. Suzuki, Y. Furusho, T. Higashi, Y. Ohnishi, S. Horinouchi, A Novel o-Aminophenol Oxidase Responsible for Formation of the Phenoxazinone Chromophore of Grixazone, *J. Biol. Chem.*, 2006, **281**, 824–833. doi: 10.1074/jbc.M505806200.

[15] A. Noguchi, T. Kitamura, H. Onaka, S. Horinouchi, Y. Ohnishi, A copper-containing oxidase catalyzes C-nitrosation in nitrosobenzamide biosynthesis, *Nat. Chem. Biol.*, 2010, **6**, 641-643. doi: 10.1038/nchembio.418.

[16] J. Jumper, R. Evans, A. Pritzel, T. Green, M. Figurnov, O. Ronneberger, K. Tunyasuvunakool, R. Bates, A. Žídek, A., Potapenko, A., Bridgland, C. Meyer, S. Kohl, A. J. Ballard, A. Cowie, B., Romera-Paredes, S. Nikolov, R. Jain, J. Adler, D. Hassabis, Highly accurate protein structure prediction with AlphaFold, *Nature*, 2021, **596**, 583–589. doi: 10.1038/s41586-021-03819-2.

[17] M. Varadi, S. Anyango, M. Deshpande, S. Nair, C. Natassia, G. Yordanova, D. Yuan, O. Stroe, G. Wood, A. Laydon, A. Zídek, T. Green, K. Tunyasuvunakool, S. Petersen, J. Jumper, E. Clancy, E., Green, A. Vora, M. Lutfi, S. Velankar, AlphaFold Protein Structure Database: massively expanding the structural coverage of protein-sequence space with high-accuracy models, *Nucleic Acids Res.*, 2022, **50**, D439–D444. doi: 10.1093/nar/gkab1061.

[18] C. K. Brown, J. F. Corbett, N. P. Loveless, Spectrophotometric studies on the protonation of hydroxy and aminophenazines in aqueous solution, *Spectrochim. Acta A*, 1979, **35**, 421–423. doi: 10.1016/0584-8539(79)80155-5.

[19] S. Fornera, P. Walde, Spectrophotometric quantification of horseradish peroxidase with *o*-phenylenediamine, *Anal. Biochem.*, 2010, **407**, 293-295. doi: 10.1016/j.ab.2010.07.034.

[20] G. A. Gamov, M. N. Zavalishin, A. Y. Khokhlova, A. V. Gashnikova, A. N. Kiselev, A. V. Zav'yalov, V. V. Aleksandriiskii, Kinetics of the Oxidation of Protocatechuic and Gallic Acids by Atmospheric Oxygen in the Presence of Laccase from *T. versicolor*, *Russ. J. Phys. Chem.*, 2020, **94**, 294–300. doi: 10.1134/S0036024420020119.

[21] W. J. Herbert, Calculating extinction coefficients for enzymatically produced *o*-quinones, *Anal. Biochem.*, 1976, **75**, 211–218. doi: 10.1016/0003-2697(76)90072-5.

[22] J. L. Muñoz, F. García-Molina, R. Varón, J. N. Rodriguez-Lopez, F. García-Cánovas, J. Tudela, Calculating molar absorptivities for quinones: Application to the measurement of tyrosinase activity, *Anal. Biochem.*, 2006, **351**, 128-138. doi: 10.1016/j.ab.2006.01.011.
